# Sex- and species-specific contribution of CD99 to T cell costimulation during multiple sclerosis

**DOI:** 10.1186/s13293-024-00618-y

**Published:** 2024-05-15

**Authors:** Ingo Winschel, Anne Willing, Jan Broder Engler, Mark Walkenhorst, Nina Meurs, Lars Binkle-Ladisch, Marcel S. Woo, Lena Kristina Pfeffer, Jana K. Sonner, Uwe Borgmeyer, Sven Hendrik Hagen, Benjamin Grünhagel, Janna M. Claussen, Marcus Altfeld, Manuel A. Friese

**Affiliations:** 1 Institute of Neuroimmunology and Multiple Sclerosis, University Medical Center Hamburg-Eppendorf, Hamburg, Germany; 2 Center of Molecular Neurobiology, University Medical Center Hamburg-Eppendorf, Hamburg, Germany; 3 Research Department Virus Immunology, Leibniz Institute of Virology, Hamburg, Germany

**Keywords:** Neuroinflammation, Sex dimorphism, T cell costimulation, Experimental autoimmune encephalomyelitis, Testosterone, X and Y chromosome, Pseudoautosomal region, Trans men, Cerebrospinal fluid, X chromosome inactivation

## Abstract

**Background:**

Differences in immune responses between women and men are leading to a strong sex bias in the incidence of autoimmune diseases that predominantly affect women, such as multiple sclerosis (MS). MS manifests in more than twice as many women, making sex one of the most important risk factor. However, it is incompletely understood which genes contribute to sex differences in autoimmune incidence. To address that, we conducted a gene expression analysis in female and male human spleen and identified the transmembrane protein CD99 as one of the most significantly differentially expressed genes with marked increase in men. CD99 has been reported to participate in immune cell transmigration and T cell regulation, but sex-specific implications have not been comprehensively investigated.

**Methods:**

In this study, we conducted a gene expression analysis in female and male human spleen using the Genotype-Tissue Expression (GTEx) project dataset to identify differentially expressed genes between women and men. After successful validation on protein level of human immune cell subsets, we assessed hormonal regulation of CD99 as well as its implication on T cell regulation in primary human T cells and Jurkat T cells. In addition, we performed in vivo assays in wildtype mice and in *Cd99*-deficient mice to further analyze functional consequences of differential CD99 expression.

**Results:**

Here, we found higher *CD99* gene expression in male human spleens compared to females and confirmed this expression difference on protein level on the surface of T cells and pDCs. Androgens are likely dispensable as the cause shown by in vitro assays and ex vivo analysis of trans men samples. In cerebrospinal fluid, CD99 was higher on T cells compared to blood. Of note, male MS patients had lower CD99 levels on CD4^+^ T cells in the CSF, unlike controls. By contrast, both sexes had similar CD99 expression in mice and *Cd99*-deficient mice showed equal susceptibility to experimental autoimmune encephalomyelitis compared to wildtypes. Functionally, CD99 increased upon human T cell activation and inhibited T cell proliferation after blockade. Accordingly, *CD99*-deficient Jurkat T cells showed decreased cell proliferation and cluster formation, rescued by *CD99* reintroduction.

**Conclusions:**

Our results demonstrate that CD99 is sex-specifically regulated in healthy individuals and MS patients and that it is involved in T cell costimulation in humans but not in mice. CD99 could potentially contribute to MS incidence and susceptibility in a sex-specific manner.

**Supplementary Information:**

The online version contains supplementary material available at 10.1186/s13293-024-00618-y.

## Plain Language Summary

The immune system protects us from bacterial and viral infections and impacts the outcome of many diseases. Thus, understanding immunological processes is crucial to unravel pathogenic mechanisms and to develop new therapeutic treatment options. Sex is a biological variable affecting immunity and it is known that females and males differ in their immunological responses. Women mount stronger immune responses leading to more rapid control of infections and greater vaccine efficacy compared to men. However, this enhanced immune responsiveness is accompanied by female preponderance and susceptibility to autoimmune diseases like systemic lupus erythematosus, rheumatoid arthritis and multiple sclerosis (MS). MS sex ratio varies around 2:1 to 3:1 with a steadily increasing incidence in female MS patients making sex one of the top risk factors for developing MS. However, the underlying biological mechanisms including sex hormones as well as genetic and epigenetic factors and their complex interplay remain largely unknown. Here, we discovered the gene and its encoded protein CD99 to be differentially expressed between women and men with men showing increased expression on many immune cell subsets including T cells. Since T cells are key contributors to MS pathogenesis, we examined the role of CD99 on T cells of healthy individuals and MS patients. We were able to identify CD99-mediated T cell regulation, which might contribute to sex differences in MS susceptibility and incidence indicating the importance to include sex as a biological variable. Of note, these differences were not reproduced in mice showing the necessity of functional research in humans.

**Supplementary Information:**

The online version contains supplementary material available at 10.1186/s13293-024-00618-y.

## Highlights

- Profiling of differential gene expression between sexes in the human immune system to delineate differential regulations that might impact immune responses.
- CD99 is genetically but not hormonally regulated on transcript and surface protein level with higher expression in men in healthy individuals as well as MS patients.
- Confirmation of CD99 as a critical co-stimulatory molecule on T cells, thus having an impact on T cell activation, proliferation and cluster formation.
- CD99 expression is elevated on T cells in the CSF compared to the blood. In male MS patients CD99 expression on CD4^+^ T cells from the CSF was lower in comparison to healthy men.
- Species difference in CD99 expression and its functional role with lack of differential CD99 expression in male and female mice as well as *Cd99* deficiency not altering experimental autoimmune encephalomyelitis.

**Supplementary Information:**

The online version contains supplementary material available at 10.1186/s13293-024-00618-y.

## Background

From epidemiological studies and clinical observations, it is known that females and males differ in their immunological responses. Women are generally able to mount stronger innate and adaptive immune responses resulting in better control of infections compared to males [1, 2]. However, this is accompanied by an increased female preponderance and susceptibility to autoimmune diseases such as systemic lupus erythematosus (SLE), rheumatoid arthritis, autoimmune liver diseases and multiple sclerosis (MS) [3, 4]. MS is a chronic inflammatory, demyelinating and neurodegenerative disease of the central nervous system (CNS) characterized by lymphocytic CNS infiltration and neuro-axonal degeneration [5, 6]. Depending on geographic regions, MS sex ratio varies between 2:1 and 3:1 [7] with a steadily increasing incidence in female MS patients making sex one of the most important risk factor for developing MS [8]. Sex hormones as well as genetic and epigenetic factors contribute to this sexual disparity. However, their complex interplay remains largely unknown.

The role of sex hormones in MS and experimental autoimmune encephalomyelitis (EAE), the animal model of MS, has been extensively studied over the past years [9, 10]. In a phase 2 multicenter trial, estriol treatment, which is an estrogen produced by the placenta during pregnancy, together with glatiramer acetate (GA), an immunomodulatory therapy, showed additional reduction in MS relapses compared to placebo plus GA [11]. Studies in EAE suggest protective mechanisms involving estrogen receptor (ER) α in peripheral immune cells [12] as well as ER β in oligodendrocytes and myeloid dendritic cells within the CNS [13]. Moreover, a possible worsening of MS disabilities can be observed with menopause [14] while evidence of effects of contraceptives have yielded conflicting results [15], suggesting the necessity of further studies on the role of female sex hormones in MS. More recently, the protective effect of androgens has been investigated. In SJL mice, mast cells produce IL-33 in response to testosterone leading to a protective phenotype in EAE by shifting the T cell response to a less pathogenic Th2 myelin-specific response [16]. Treatment with daily testosterone (androgel) in ten male MS patients suggested a neuroprotective effect [17].

While it is generally not possible to study the genetic status independently of sex hormones in humans, studies in people with sex chromosome disorders show an increased risk of autoimmune diseases like SLE and MS in men with Klinefelter’s syndrome (XXY) and an underrepresentation of women with Turner’s syndrome (45,X) among SLE patients [18, 19]. To study the effect of sex chromosomes independently of hormones, Four Core Genotype (FCG) mice were used, in which the *Sry* was deleted from the Y chromosome and transferred to an autosome. Mice with XX sex chromosome background demonstrated a more proinflammatory phenotype and greater susceptibility to EAE and lupus compared with XY mice [20], indicating that X-chromosomal encoded genes show an independent risk of hormonal regulation. While the Y chromosome harbors 106 protein-encoding genes [21], the X chromosome encodes more than 800 protein-coding genes, from which several contribute to sex-based differences in immune responses. Among those immune genes are receptors like IL-2 receptor γ-chain (IL-2RG), toll-like receptor 7 (TLR7) and proteins involved in transcriptional and translational control like forkhead box P3 (FOXP3) as well as proteins related to immune response like CD40 ligand (CD40L) and CD99 [22]. Regarding (epi)genetic factors, the presence of the X and Y chromosome and the mechanism of X chromosome inactivation (XCI) are of particular importance. Recently, two studies investigated the role of the Y chromosome in colorectal and bladder cancer. While the loss of the entire Y chromosome (LOY) raises the risk for bladder cancer by evading detection from immune cells [23], another study discovered a detrimental role for the Y chromosome gene *KDM5D* in colorectal cancer [24]. Similarly, the X-linked histone demethylase *Kdm6a*, capable of escaping XCI and showing sexually dimorphic expression, ameliorated the clinical disease course and reduced neuropathology in EAE when deleted in T cells [25].

T cell infiltration from the periphery across the blood brain barrier (BBB) promotes inflammation, demyelination and neurodegeneration leading to lesions in the CNS, which are hallmarks of MS [5]. It is thought that these T cells recognize CNS autoantigens, as they escaped central tolerance, are activated at peripheral sites and traffic to the CNS, where they get reactivated by innate CNS-resident immune cells. The cell surface receptor CD99 has been suggested to be important in transmigration of immune cells through endothelial layers including the BBB [26–28]. The 32-kDa transmembrane protein CD99 is broadly expressed in many human cell types including leukocytes and endothelial cells [26, 29]. It is encoded by the gene *MIC2*, which is located at the very edge of the pseudoautosomal region of the X and Y chromosome. This region is known to escape XCI, which also applies for CD99 [30]. However, this escape might not always happen to a full extent leading to a differential expression in females and males [31]. Accordingly, individuals with Turner syndrome (45,X), a unique sex chromosome aneuploidy, show a decreased *CD99* expression compared to euploidic females (46,XX) and males (46,XY) [32, 33]. Among many different functions, CD99 might participate in T cell activation [34–36].

Notably, genome-wide association studies (GWAS) in MS have only focused on autosomal chromosomes and their contribution to MS susceptibility. Although recent GWAS partially included analysis of sex chromosomes, no protein-coding X- or Y-linked risk variants were detected [37, 38]. This was possibly due to exclusion of SNPs from the X and Y chromosome as they did not pass quality control criteria and limited statistical power for both sex chromosomes. Having these limitations in mind, together with the observation that genetic variation accounts for approximately 30% of the overall disease risk [39], made us hypothesize that there are undetected gene expression differences between females and males that could explain part of the sexual dimorphism in incidence of MS and other autoimmune diseases.

Here, we show that immune cells differ in CD99 surface expression between women and men in the peripheral blood and this difference is retained in T cells in the CSF. However, MS patients show overall decreased CD99 expression on T cells in the CSF compared to healthy controls and this is mainly driven by decreased expression in MS males compared to healthy males. Moreover, we show that CD99 impacts T cell activation, proliferation and cluster formation in diverse assays ranging from primary human T cells to genetically modified T cell lines. Eventually, we demonstrate that increased CD99 expression in males is not primarily mediated by testosterone, thus enforcing the importance of transcriptional CD99 regulation from the Y chromosome. In mice, we cannot detect a sex-specific difference in CD99 expression and also genetic ablation or reduced expression in the mouse model were insufficient to recapitulate human functional sex-specific differences. However, consistent sex-specific differential expression of the CD99 molecule with functional relevance in T cells underlines the great importance of sex as biological variable in biomedical research that must be considered for individualized therapy in the context of autoimmunity.

## Methods

### Study cohorts

MS patients, NND patients and healthy individuals were recruited through the MS day clinic and the Department of Neurology, University Medical Center Hamburg-Eppendorf (UKE). The study was approved by the local ethics committee (Hamburger Patienteninformationssystem Multiple Sklerose-HAPIMS, Ethikkommission der Ärztekammer Hamburg, registration number PV4405) and informed consent was obtained from all patients and healthy individuals. These patients were not on any immunomodulatory medication at the time of sampling. These cryopreserved samples were collected in the biobank of the UKE Institute of Neuroimmunology and Multiple Sclerosis (INIMS). Trans men were recruited through the medical practice amedes Medizinisches Versorgungszentrum Hamburg GmbH. The study was approved by the local ethics committee (Ethikkommission der Ärztekammer Hamburg, registration number PV5245) and informed consent was obtained from all individuals. These cryopreserved samples were collected at the Research Department Virus Immunology, Leibniz Institute of Virology, Hamburg. Study participants were screened for chronic infections, autoimmune and metabolic diseases and cancers by medical history and blood tests. Participants received 1000 mg testosterone undecanoate i.m. (Nebido, Jenapharm, Jena, Germany) injections. After the first testosterone injection, the second injection was scheduled 6 weeks later, followed by a steady rhythm of 3 months. Further characteristics of the cohorts are detailed in Tables S1–3.

### GTEx analysis

Gene expression data of spleen samples from 87 women and 154 men was downloaded from the Genotype-Tissue Expression (GTEx) project at https://gtexportal.org (GTEx_Analysis_v8). Raw RNA-seq read counts were analyzed for differential expression between sexes using DESeq2_1.34.0 (Table S6). Genes with exclusive localization on one of the sex chromosomes were excluded. According to our cut off criteria (false discovery rate-adjusted *P* value < 0.05; absolute log2 fold change > 0.2), 408 differentially expressed genes were identified (153 higher in women, 255 higher in men; Table S7). To test for the influence of sex hormone levels on gene expression, premenopause samples (38 women, 63 men, age ≤ 49 years) were compared to postmenopause samples (49 women, 91 men, age ≥ 50 years) separately in both sexes using DESeq2_1.34.0 (Table S8, S9). Plotting of the gene expression data was performed using ggplot2_3.4.2 and tidyheatmaps_0.1.0 (https://github.com/jbengler/tidyheatmaps).

### PBMC isolation and cryopreservation

Blood was collected in EDTA coated tubes (Sarstedt) and processing of blood samples was started immediately upon receipt of blood samples in the laboratory. For MS patients, NND patients and healthy individuals PBMCs were isolated by a 30 min gradient centrifugation at 860 ×*g* using Biocoll Separating Solution (Biochrom), followed by two washing steps. Cells were then cryopreserved in RPMI medium (PAN-Biotech) with 25% heat-inactivated fetal calf serum (FCS, Biochrom) and 10% (v/v) dimethyl sulfoxide (DMSO, AppliChem) and stored in liquid nitrogen until further analysis. For trans men PBMCs were isolated by a 30 min gradient centrifugation at 500 ×*g* using Lymphocyte Separation Media (Capricorn), followed by two washing steps. After lysis of erythrocytes with ACK Lysing Buffer (Lonza), cells were then cryopreserved in heat-inactivated fetal bovine serum (FBS) supplemented with 10% (v/v) dimethyl sulfoxide (SigmaAldrich) and stored in liquid nitrogen until further analysis.

### Flow cytometry

#### Surface staining

Staining of surface marker antigens was always performed at 4 °C using monoclonal fluorochrome labeled antibodies. In the case of whole blood samples subsequently erythrocytes were lysed and lymphocytes fixed using BD FACS™ Lysing Solution. PBMCs and lymphocytes derived from CSF were fixed with BD Cytofix™ Fixation Buffer after staining prior to analysis. Surface marker antibodies used in this study are listed in Table S4.

#### Absolute cell quantification

Absolute cell counts were quantified by using Precision Count Beads™ (BioLegend).

#### Sample analyses

All samples were acquired on a BD FACS LSR II analyzer or FACSymphony A3 (BD Biosciences). Flow cytometry-based cell sorting was performed on a FACSAria III cell sorter (BD Biosciences). Data was analyzed with FlowJo software (Version 10.8.1, BD Biosciences).

### Human T cell proliferation

Cryopreserved PBMCs from healthy women and men were thawed and CD3^+^ T cells were isolated from single-cell suspension using the Human Pan T Cell Isolation Kit (Miltenyi) according to the manufacturer’s protocol and labelled with CFSE (CellTrace™ CFSE Cell Proliferation Kit, ThermoFisher) according to the manufacturer’s protocol. CD3^+^ T cells were seeded at a density of 25,000 cells per well in an anti-CD3 (0.5 µg/ml, clone OKT3, BioLegend) coated 96-well plate. Cells were supplemented with soluble anti-CD28 (5 µg/ml, clone CD28.2, BioLegend) and anti-CD99 (5 µg/ml, clone HCD99, BioLegend) or respective isotype control (5 µg/ml, clone MOPC-173, BioLegend). Proliferation was tracked by cluster formation in the IncuCyte^®^ for 7 days.

### Human T cell activation

Cryopreserved PBMCs from healthy women were thawed and seeded at a density of 2 × 10^5^ cells per well in an anti-CD3 (10 µg/ml, clone OKT3, BioLegend) coated 96-well plate. Cells were supplemented with soluble anti-CD28 (2.5 µg/ml, clone CD28.2, BioLegend) and anti-CD99 (10 µg/ml, clone hec2, BioLegend) or respective isotype control (10 µg/ml, clone MOPC-21, BioLegend). Samples were incubated for 72 h at 37 °C and 5% CO_2_, stained with antibodies listed in Table S4 and analyzed by flow cytometry after 2, 24, 48 and 72 h.

### In vitro testosterone treatment

For testosterone treatment, we used RPMI 1640 Medium, without L-glutamine, without phenol red (Capricorn) supplemented with 1% GlutaMAX™ Supplement (Gibco), 1% Pen Strep (Gibco) and 5% charcoal stripped FBS Standard (PAN Biotech). For charcoal stripping of FBS, 2 g dextran-coated charcoal (DCC, Merck) were added to 100 ml FBS and incubated on a shaker overnight at 4 °C followed by centrifugation and filtration. Cryopreserved PBMCs from male healthy individuals were thawed and seeded at a density of 2 × 10^5^ cells per well in an anti-CD3 (10 µg/ml, clone OKT3, BioLegend) coated 96-well plate and supplemented with soluble anti-CD28 (2.5 µg/ml, clone CD28.2, BioLegend) or left unstimulated. Subsequently, testosterone (3, 30 and 300 ng/ml, Sigma Aldrich) or 5α-dihydrotestosterone (0.3, 3 and 30 ng/ml, SigmaAldrich) was added. Samples were incubated for 48 h at 37 °C and 5% CO_2_, stained with antibodies listed in Table S4 and analyzed by flow cytometry after 2, 24 and 48 h.

### Mice

C57BL/6J WT and C57BL/6J-CD99^em2−8/Hhtg^ (*Cd99-deficient*) were kept under specific pathogen-free conditions in the animal facility of the ZMNH, University Medical Centre Hamburg-Eppendorf. 8–12-week-old mice were used for experiments. All animal experimental procedures were in accordance to international and national animal welfare guidelines. Ethical approvals were obtained from the State Authority of Hamburg, Germany (Approval no. 083/19 and 107/21).

### Generation of *Cd99*-deficient mice

To generate *Cd99*-deficient mice, the second coding exon was targeted by the CRISPR/Cas9 genome editing system. A single sgRNA was designed using the CRISPOR design tool (http://crispor.tefor.net) [40]. The template for transcription with the targeting sequence (GCGAGTGACGACTTCAACCT) was generated by fill-in reaction with Klenow DNA Polymerase (Thermo Fisher Scientific). Transcription was performed using the HiScribe™ *T7* High Yield RNA Synthesis Kit (#E2040S, New England Biolabs), with subsequent purification of the transcript with the MEGAClear™ Transcription Clean-Up Kit (#AM1908, Thermo Fisher Scientific), both according to the manufacturer’s instructions. The sgRNA (600 ng/µL) and Cas9 protein (Alt-R^®^ S.p. Cas9 Nuclease V3, #1,081,058, Integrated DNA Technologies (IDT)) (500 ng/µL) in Gibco™ Opti-MEM™ (Thermo Fisher Scientific) were electroporated into one-cell-stage embryos derived from superovulated C57BL/6J mice using the NEPA 21 electroporator (Nepa Gene) [41]. Embryos were implanted into F1 foster mothers (C57BL/6 × CBA) and the resulting offspring was analyzed by PCR amplification on genomic tail DNA using primers F (5′-CGG GGC CCG GAT TGG ATG TAA ATG CTG-3′) and R (5′- AGA GCC CCG GGT ATG TAA ATG ACT C-3′) and subsequent Sanger sequencing. For our experiments, we used animals with an insertion of 2 bp in exon 2, which resulted in CD99 protein deficiency. These offspring were analyzed by separate PCRs with either WT allele-specific forward primer (CCT CAG CGA GTG ACG ACT TCA ACC) or mutant allele-specific forward primer (CCT CAG CGA GTG ACG ACT TCA AAA CC) and common reverse primer (CCC AGA GCC CCG GGT ATG TAA ATG ACT C). WT specific PCR resulted in a PCR product of 180 bp and mutant specific PCR resulted in a product of 182 bp.

### Mouse T cell proliferation

Lymph nodes (axillary, brachial, inguinal) and spleen from homo- and heterozygous *Cd99*-deficient and C57BL/6 WT mice were collected in ice-cold PBS. CD3^+^ T cells were isolated from single-cell suspension using the MojoSort™ Mouse CD3 T Cell Isolation Kit (BioLegend) according to the manufacturer’s protocol and labelled with CFSE (CellTrace™ CFSE Cell Proliferation Kit, ThermoFisher) according to the manufacturer’s protocol. CD3^+^ T cells were seeded at a density of 3 × 10^5^ cells per well in an anti-CD3 (1 µg/ml, clone 145-2CL11, BioLegend) coated 96-well plate. Cells were supplemented with soluble anti-CD28 (0.5 µg/ml, clone 37.51, BioLegend) and IL-2 (20 IU/ml, Peprotech). Samples were incubated for 72 h at 37 °C and 5% CO_2_, stained with antibodies listed in Table S4 and analyzed by flow cytometry.

### EAE induction

#### Active immunization

Mice were immunized subcutaneously with 200 μg of MOG_35–55_ peptide (Peptide and elephants) in complete Freund’s adjuvant (CFA) containing 2 mg/ml *Mycobacterium tuberculosis* (BD Bioscience). Additionally, 200 ng pertussis toxin (Merck Millipore) in PBS were injected intraperitoneally (i.p.) on the day of immunization and 48 h later.

#### EAE scoring

Weight and clinical signs of disease were scored daily starting at day 7 by the following system: 0, no clinical deficits; 1, tail weakness; 2, hind limb paresis; 3, partial hind limb paralysis; 3.5, full hind limb paralysis; 4, full hind limb paralysis and forelimb paresis; 5, premorbid or dead. Animals reaching a clinical score of ≥ 4 or having more than 25% body weight loss (from starting weight) were sacrificed according to regulations of the local Animal Welfare Act. Scoring of EAE experiments was performed blinded for the genotype. Mice without any disease symptoms 20 days post immunization were excluded from the analyses. To minimize cage-specific effects, experimental groups were mixed within cages and littermates were used as control animals.

### Immune cell isolation

#### Spleen and LN

Inguinal, brachial and axillary lymph nodes as well as spleen samples were homogenized through a 70 µm cell strainer and washed with PBS (300 ×*g*, 10 min, 4 °C). Splenic cells were resuspended in erythrocyte lysis buffer (10 mM potassium bicarbonate, Merck Millipore; 0.15 M ammoniochloride, Merck Millipore; 0.1 mM Na_2_EDTA, Thermo Fisher Scientific; in ddH_2_O; pH 7.4) and incubated for 5 min on ice to lyse red blood cells before it was stopped with PBS.

#### CNS

Before the CNS was taken, mice were intracardially perfused with 10 ml PBS. The CNS was minced with a razor blade, digested for 45 min at 37 °C in a shaking water bath (1 mg/ml Collagenase A, Roche; 0.1 mg/ml DNaseI, Merck Millipore; in RPMI-1640 medium, PAN Biotech) and subsequently homogenized through a 70 µm cell strainer. After washing with PBS, immune cells were isolated by percoll gradient centrifugation (30%/78% 1.13 g/ml, GE Healthcare) at 2500 rpm, 30 min, 4 °C, w/o brake, harvested from the interphase and washed twice with PBS.

### Jurkat T cell proliferation

WT Jurkat T cells, *CD99*-deficient Jurkat T cells or transduced Jurkat T cells were fluorescently labelled (CellTrace™ Violet Cell Proliferation Kit, ThermoFisher) according to the manufacturer’s protocol. Cells were seeded at a density of 3 × 10^5^ cells per well in a 12-well plate. Samples were incubated for 96 h at 37 °C and 5% CO_2_ and analyzed by flow cytometry after 0, 24, 48, 72 and 96 h. Jurkat T cells are derived from a male donor and harbor X and Y chromosomes.

### Vector construction and lentiviral production

To insert human CD99 (hCD99) into a lentiviral vector harboring a CMV promoter, we first performed PCR using Primer_f_1 and Primer_r_1 from a human CD99 gene ORF cDNA clone expression plasmid (Biozol). Restriction and ligation were performed using BamHI and XbaI restriction sites. To generate hCD99 CRISPR-KO cells, we used four guide RNAs (Oligo_f/r_1, Oligo_f/r_2, Oligo_f/r_3, Oligo_f/r_4) targeting different protein coding exonic regions of hCD99 that were annealed and inserted using Esp3I restriction sites into the Lenti-Cas9-gRNA-GFP. Lenti-Cas9-gRNA-GFP was a gift from Jason Sheltzer (Addgene #124,770; http://n2t.net/addgene:124770; RRID: Addgene_124770). Jurkat T cells were either transfected by Lipofectamine 2000 (ThermoFisher) according to the standard protocol or by lentiviral transduction (see below for detailed description). GFP-expressing transfected cells were identified and sorted by flow cytometry using a FACSAria III cell sorter (BD Biosciences). Genetic KO was confirmed using flow cytometry and Sanger sequencing. To produce lentiviruses, we first transfected HEK293T cells with 10 µg expression plasmid, 10 µg pMDLg/pRRE, 5 µg pRSV-Re, 2 µg pMD2.G. pMDLg/pRRE was a gift from Didier Trono (Addgene #12,251; http://n2t.net/addgene:12251; RRID: Addgene_12251). pRSV-Rev was a gift from Didier Trono (Addgene #12,253; http://n2t.net/addgene:12253; RRID:Addgene_12253). pMD2.G was a gift from Didier Trono (Addgene #12,259; http://n2t.net/addgene:12259; RRID: Addgene_12259). Briefly, HEK293T cells were seeded out with an 80% confluency in DMEM with glutamine and high glucose (ThermoFisher), the next day the plasmids were mixed in 1 × HEPES buffered saline (HBS) and 125 mM CaCl_2_ and were applied to the HEK293T cells for 6 h. Subsequently, medium was changed and after 48 h the supernatant was filtered through a 0.45 µm PES filter, immediately snap frozen and stored at − 80 °C. Primers, oligonucleotides, and the respective restriction sites are listed in Table S5.

### Statistical analysis

Bar graphs represent mean ± standard error of the mean (SEM). Statistical analyses were performed using Prism 9 software (GraphPad Software). Normal distribution was tested using Kolmogorov–Smirnov or Shapiro–Wilk test. Significant results are indicated by *P < 0.05, **P < 0.01, ***P < 0.001.

## Results

### Increased CD99 expression by male immune cells

**Fig. 1:**
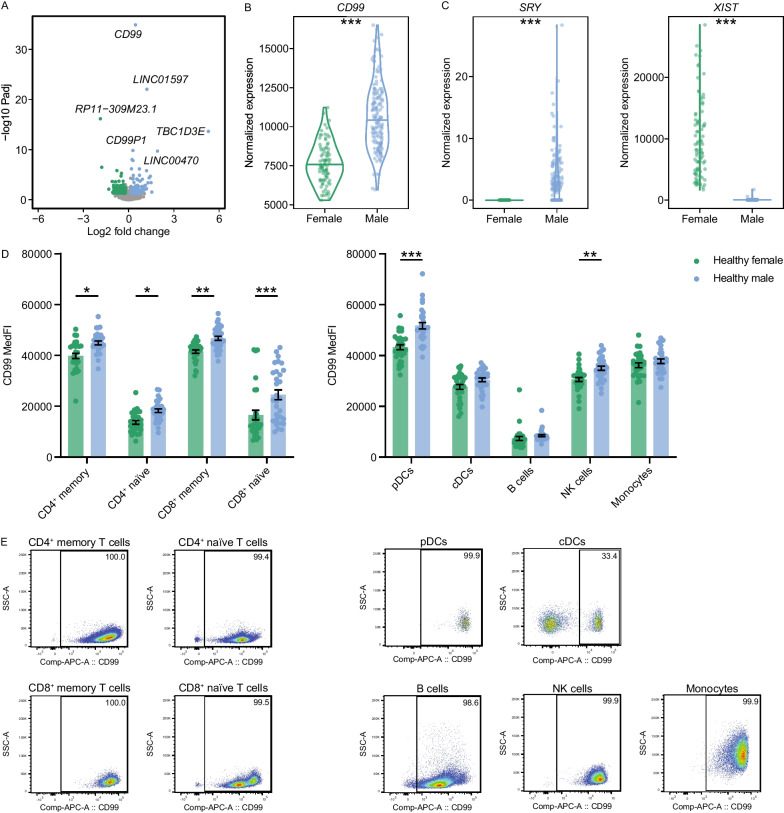
Increased CD99 expression in immune cells from men. **A** Sex-specific differentially expressed (pseudo)autosomal genes in spleen samples (*n* = 87 women and 154 men) from the Genotype-Tissue Expression (GTEx) dataset. **B**, **C** Corresponding mRNA expression levels of *CD99, SRY* and *XIST* in spleen. **D** CD99 surface protein expression on subsets of cryoconserved peripheral blood mononuclear cells (PBMCs) from male (*n* = 30) and female (*n* = 30) healthy individuals as analyzed by flow cytometry. Cell subsets were identified as following: CD4^+^ and CD8^+^ memory (gated as living CD3^+^-CD4^+^/CD8^+^-CD45RA^–^ cells) and naïve (gated as living CD3^+^-CD4^+^/CD8^+^-CD45RA^+^ cells) T cells; pDCs (gated as living CD45^+^-Lineage(CD3, CD14, CD19, CD20, CD56)^–^-HLA-DR^+^-CD11c^–^-CD123^+^-CD304^+^ cells); cDCs (gated as living CD45^+^-Lineage(CD3, CD14, CD19, CD20, CD56)^–^-HLA-DR^+^-CD11c^+^ cells); B cells (gated as living CD45^+^- CD19^+^-CD20^+^ cells); NK cells (gated as living CD45^+^- CD19^–^-CD20^–^-CD3^–^-CD14^–^-CD56^+^ cells) and monocytes (gated as living CD45^+^- CD19^–^-CD20^–^-CD3^–^-CD14^+^ cells). **E** Representative flow cytometry plots of CD99 surface expression on immune cells shown in (**D**). Data are shown as violin plots including median. Statistics: **A**–**C** DESeq2 false discovery rate-adjusted *P* value; **D** two-way ANOVA with Tukey post-hoc; *P < 0.05; **P < 0.01; ***P < 0.001

To identify genes that are differentially regulated in women and men and might contribute to sex-specific differences in autoimmunity, we conducted an unsupervised and systematic gene expression analysis. We made use of a publicly available dataset compiled by The Genotype Tissue Expression (GTEx) project [42], that includes information about the donor’s sex, and analyzed mRNA expression in the spleen. Of note, we focused on genes, which are not exclusively located on the X or Y chromosome, thus mainly focusing on (pseudo)autosomal differences. With our cut-off criteria (absolute log2 fold change > 0.2; *padj* < 0.05), we identified 408 differentially expressed genes (DEGs) between women and men in the spleen, of which 153 were higher in women and 255 were higher in men (Fig. 1A; Figure S1A, B). These DEGs included protein-coding genes (e.g., *TBC1D3E*, *DHRSX* and *ZBED1*) as well as pseudogenes (e.g., *CD99P1* and *MTND1P23*), long non-coding RNAs (e.g., *LINC01597* and *LINC00470*) and unspecific transcripts (e.g., *RP11-309M23.1*). Protein-coding genes that were increased in males included *TBC1D3E*, *DHRSX* and *ZBED1*, whereas *NLRP7*, *MPP6* and *PRSS16* were increased in women. However, the candidate with the highest significance according to the adjusted *P* value was *CD99* (absolute log2 fold change = 0.47; *padj* = 1.34 × 10^–35^), showing an increased expression in males (Fig. 1B). As internal validation of the dataset, we detected *XIST* expression only in women and *SRY* only in males (Fig. 1C). *XIST* encodes for the X inactive specific transcript and should therefore only be expressed in women, while *SRY* encodes for the sex-determining region Y, which is located on the Y chromosome. In order to investigate, whether sex-specific expression differences of CD99 on mRNA level translate to differential expression on protein level and in particular on functionally relevant surface expression level, we performed flow cytometry analyses of cryoconserved PBMCs from healthy individuals (HI) with *n* = 30 individuals per group. Indeed, surface expression of CD99 was significantly higher in male HI on CD4^+^ and CD8^+^ memory and naïve T cells as well as on pDCs and NK cells (Fig. 1D, E; Figure S2A–C). We did not observe any significant differences in cDCs, B cells and monocytes. Together, we detected CD99 to be markedly higher expressed in male spleens on transcript level and on CD4^+^ and CD8^+^ memory and naïve T cells, pDCs and NK cells on surface protein level.

### Testosterone does not regulate CD99 expression

**Fig. 2:**
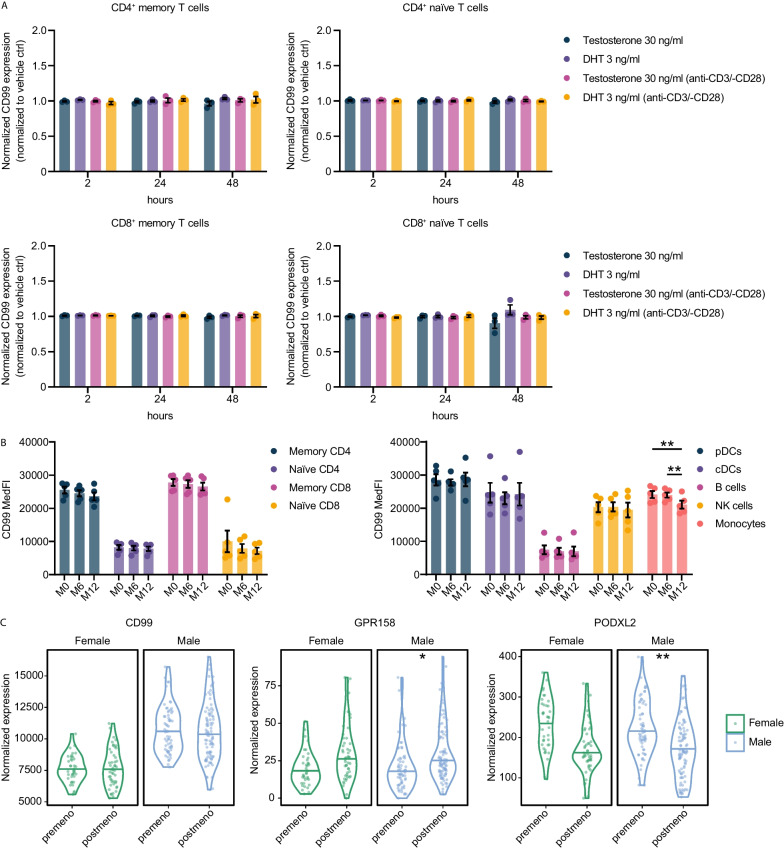
CD99 expression is independent of sex hormones. **A** CD99 surface expression of T cells (*n* = 3 men). Cryopreserved PBMCs were either stimulated with anit-CD3 and anti-CD28 mAbs or left unstimulated and treated with either testosterone (30 ng/ml) or dihydrotestosterone (DHT; 3 ng/ml). **B** CD99 surface protein expression on subsets of cryoconserved peripheral blood mononuclear cells (PBMCs) from trans men individuals (*n* = 5) receiving testosterone treatment as analyzed by flow cytometry at baseline (month 0, M0), after 6 months (M6) or 12 months (M12) of treatment. Cell subsets were identified as following: CD4^+^ and CD8^+^ memory (gated as living CD3^+^-CD4^+^/CD8^+^-CD45RA^–^ cells) and naïve (gated as living CD3^+^-CD4^+^/CD8^+^-CD45RA^+^ cells) T cells; pDCs (gated as living CD45^+^-Lineage(CD3, CD14, CD19, CD20)^–^-HLA-DR^+^-CD11c^–^-CD123^+^ cells); cDCs (gated as living CD45^+^-Lineage(CD3, CD14, CD19, CD20)^–^-HLA-DR^+^-CD11c^+^ cells); B cells (gated as living CD45^+^-CD19^+^-CD20^+^ cells); NK cells (gated as living CD45^+^-CD19^–^-CD20^–^-CD3^–^-CD14^–^ HLA-DR^—^CD56^+^ cells) and monocytes (gated as living CD45^+^- CD19^–^-CD20^–^-CD3^–^-CD14^+^ cells). **C** mRNA expression levels of *CD99, GPR158 and PODXL2* in pre- or postmenopausal samples of previously analyzed GTEx dataset in women and men. Data are shown as mean ± SEM (**A**, **B**) and violin plots including median (**C**). Statistics: **A**, **B** two-way ANOVA with Tukey post-hoc; **C** DESeq2 false discovery rate-adjusted *P* value; *P < 0.05; **P < 0.01; ***P < 0.001

Although CD99 expression is genetically regulated by its localization on both sex chromosomes and the ability to escape XCI, we next tested whether CD99 surface expression could additionally be regulated by sex hormones [1, 2]. To probe whether increased CD99 levels in men could be attributed to the influence of testosterone, we first treated T cell receptor (TCR)-stimulated and unstimulated PBMCs with different concentrations of testosterone and dihydrotestosterone, its most potent bioactive form, for 48 h. Neither testosterone nor dihydrotestosterone had an influence on CD99 surface expression in this in vitro setting (Fig. 2A; Figure S3A, B). Second, to corroborate these findings ex vivo, we analyzed CD99 surface expression of cryoconserved PBMCs from trans men, who received gender-affirming hormone therapy (GAHT) with intramuscular (i.m.) testosterone treatment. Total testosterone as well as bioavailable testosterone serum concentrations reached male reference ranges [43] over the time of observation (Figure S3C). When comparing baseline levels (month 0, M0) to protein levels after 6 months (M6) or 12 months (M12) of treatment, we did not observe changes of CD99 surface expression upon testosterone treatment in any immune cell subset (T cells, DCs, B cells and NK cells) but in monocytes, where we saw a decrease after 6 and 12 months (Fig. 2B, Figure S4A, B). Third, we re-analyzed our GTEx dataset separating samples into pre- (≤ 49 years) and postmenopausal (≥ 50 years) to ask whether sex hormone level decline with age is associated with changes in CD99 expression levels. While there was no change in expression levels for *CD99*, other representative genes like *GPR158* or *PODXL2* were regulated, showing overall analysis validity (Fig. 2C). Taken together, these data indicate that testosterone is likely dispensable as a driver of sex-specific CD99 expression levels.

### CD99 increase in the CSF and partial loss of sex-specific differences driven by male MS patients

**Fig. 3:**
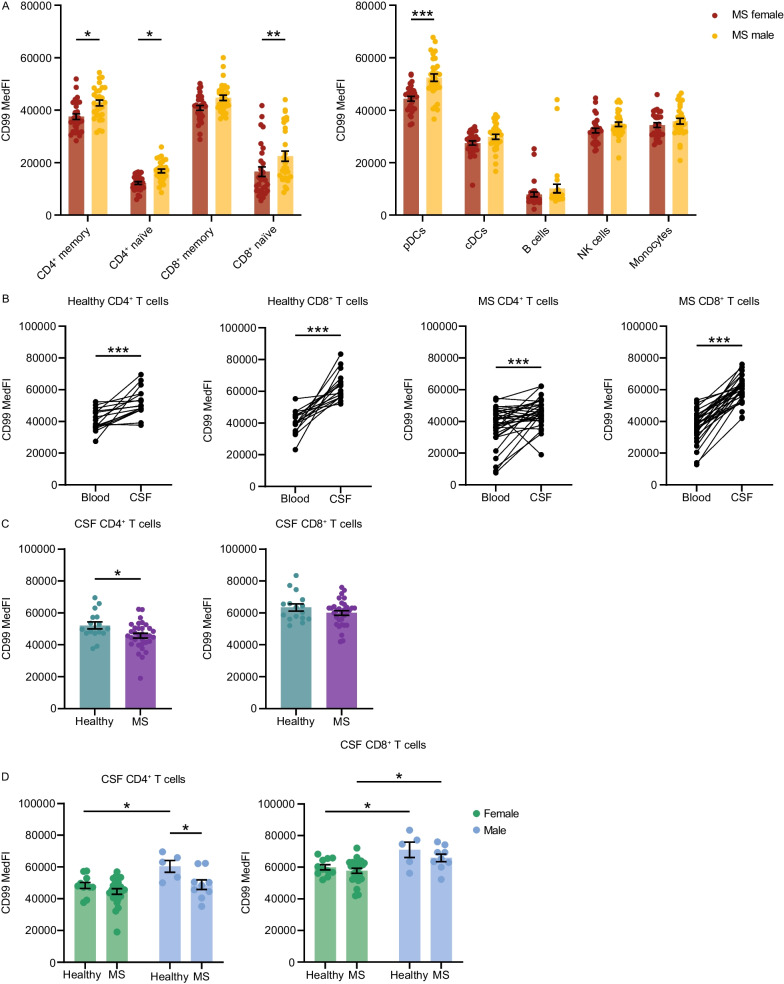
CD99 reduction on T cells in the CSF of male MS patients. **A** CD99 surface protein expression on subsets of cryoconserved peripheral blood mononuclear cells (PBMCs) from male (*n* = 30) and female (*n* = 30) MS patients as analyzed by flow cytometry. Cell subsets were identified as following: CD4^+^ and CD8^+^ memory (gated as living CD3^+^-CD4^+^/CD8^+^-CD45RA^–^ cells) and naïve (gated as living CD3^+^-CD4^+^/CD8^+^-CD45RA^+^ cells) T cells; pDCs (gated as living CD45^+^-Lineage(CD3, CD14, CD19, CD20, CD56)^–^-HLA-DR^+^-CD11c^–^-CD123^+^-CD304^+^ cells); cDCs (gated as living CD45^+^-Lineage(CD3, CD14, CD19, CD20, CD56)^–^-HLA-DR^+^-CD11c^+^ cells); B cells (gated as living CD45^+^- CD19^+^-CD20^+^ cells); NK cells (gated as living CD45^+^- CD19^–^-CD20^–^-CD3^–^-CD14^–^-CD56^+^ cells) and monocytes (gated as living CD45^+^- CD19^–^-CD20^–^-CD3^–^-CD14^+^ cells). **B** CD99 surface expression on freshly isolated T cells in peripheral blood and CSF of non-neuroinflammatory controls (*n* = 16) and MS patients (*n* = 33) as analyzed by flow cytometry. **C** Comparison of CD99 expression levels in the same set of non-inflammatory controls and MS patients. **D** Sex-specific analysis of CD99 surface expression in the same set of healthy individuals (*n* = 11 females, 5 males) and MS patients (*n* = 24 females, 9 males). Data are shown as mean ± SEM. Statistics: **A** two-way ANOVA with Tukey post-hoc; **B** Wilcoxon matched-pairs signed rank test; **C** Mann Whitney test; **D** two-way ANOVA with Sidak’s post-hoc; *P < 0.05; **P < 0.01; ***P < 0.001

Sex-specific differential surface expression of CD99 on T cells and pDCs could possibly contribute to higher female preponderance in autoimmune diseases. To probe whether surface expression changes were altered in autoimmunity, we performed flow cytometry analyses of cryoconserved PBMCs from MS patients with *n* = 30 individuals per group. Notably, the sex-specific expression pattern observed in HI was equally detectable in CD4^+^ memory and naïve T cells, CD8^+^ naïve T cells and pDCs in MS patients and there were no expression differences between HI and MS patients (Fig. 3A, Figure S5A). Hence, differential CD99 expression is not regulated in this autoimmune disease in the peripheral blood, but could still mediate functional differences of T cells and pDCs with relevance for the disease pathogenesis and perpetuation. Such as, CD99 has been proposed to be involved in BBB transmigration [28, 44]. Therefore, we next analyzed CD99 surface protein expression on T cells from the CSF of MS patients (*n* = 24 females, 9 males) and of patients with non-neuroinflammatory diseases (NND patients;* n* = 11 females, 5 males). As CD99 is mainly expressed on memory T cells (Fig. 1D; Fig. 3A; Figure S5B, C) and as naïve T cells are basically absent from the CSF (Figure S5D), we quantified CD99 expression on non-naïve T cells and compared expression levels in the CSF to the blood of the same donor drawn at the same day. Indeed, CD99 expression was markedly increased on non-naïve CD4^+^ T cells and CD8^+^ T cells in the CSF compared to the peripheral blood in both patient cohorts (Fig. 3B), likely due to the increased ability of CD99 high expressing T cells to transmigrate to the CNS. Of note, we detected an overall lower CD99 surface expression on non-naïve CD4^+^ T cells in the CSF of MS patients compared to NND patients (Fig. 3C**).** Stratifying CD99 expression on CSF T cells by sex revealed that lower CD99 expression levels in the MS cohort were solely driven by lower expression in male MS patients, while in women there was no difference between MS and NND patients (Fig. 3D). This lower CD99 expression on T cells in male MS patients led to a loss of the sex-specific expression pattern in the CSF on CD4^+^ T cells and to a lesser pronounced sex-specific difference in CD8^+^ T cells in disease context.

### *Cd99*-deficient mice are insufficient to reproduce sex-dosage phenotype in humans

**Fig. 4:**
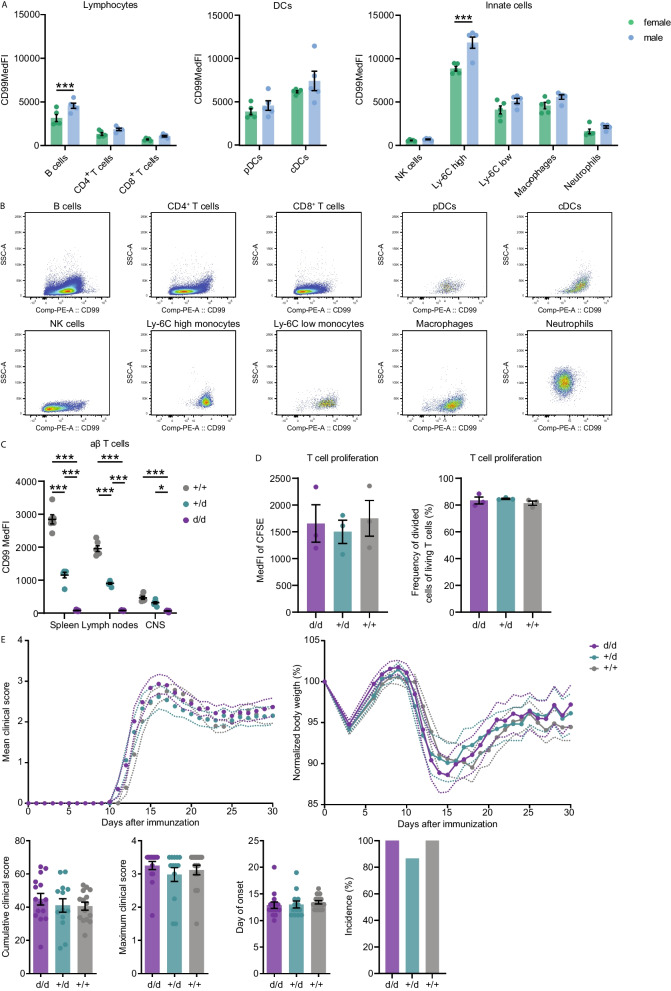
*Cd99*-deficient mice show no difference in immune cell function. **A** CD99 surface expression of immune cells in C57BL/6J WT mice (*n* = 5 females, *n* = 5 males). **B** Representative flow cytometry plots of CD99 surface expression on immune cells shown in (**A**). **C** CD99 surface expression of T cells in homozygous *Cd99*^–/–^, heterozygous *Cd99*^–/–^ and WT littermates (*n* = 4 female d/d, *n* = 2 male d/d,* n* = 3 female + /d, *n* = 3 male + /d,* n* = 3 female +/+, *n* = 3 male +/+). **D** Cell proliferation assay with isolated T cells from homozygous *Cd99*^–/–^, heterozygous *Cd99*^–/–^ and WT littermates (*n* = 2 females and 1 male per group). **E** EAE course in homozygous *Cd99*^–/–^, heterozygous *Cd99*^–/–^ and WT littermates after active immunization against MOG_35-55_ peptide (*n* = 8 female d/d, *n* = 7 male d/d,* n* = 6 female + /d, *n* = 7 male + /d,* n* = 8 female +/+, *n* = 7 male + / +). Data are shown as mean ± SEM. Statistics: **A** two-way ANOVA with Sidak’s post-hoc; **C** two-way ANOVA with Tukey post-hoc; **D** Kruskal–Wallis test with Dunn post-hoc; **E** Mann–Whitney U test; *P < 0.05; **P < 0.01; ***P < 0.001

The cause of reduced CD99 expression on T cells in the CSF in male MS patients is difficult to infer and functional consequences are challenging to address in the absence of experimental manipulation in humans. Therefore, we next set out to establish a mouse model to investigate functional consequences of CD99 expression in the CNS. As a first step, we analyzed CD99 surface expression on immune cell subsets in C57BL/6J wildtype (WT) mice. While CD99 levels were increased in B cells and Ly-6C high monocytes of male mice, all other subsets including T cells did not display sex-specific expression patterns and thus failed to reproduce results from human PBMCs (Fig. 4A, B; Figure S6A–C). Given the solely genetic and not hormonal regulation of sex-specific CD99 expression patterns in humans and the fact that in mice CD99 is encoded on an autosomal chromosome, this result is not surprising and corroborates our previous findings. To still use mice to address functional consequences of altered CD99 expression, we generated *Cd99*-deficient mice with the idea that the heterozygous knockout mice would show lower CD99 expression, thus reflecting decreased levels that we observed in women. Indeed, we confirmed the absence of CD99 in T cells from the spleen, lymph nodes and from the CNS of homozygous knockout mice and a decreased expression in heterozygous knockout mice (Fig. 4C). Since CD99 has been shown to participate in T cell proliferation [45], we performed a dye-based cell proliferation assay with T cells from these three genotypes, but did not see any difference in cell proliferation (Fig. 4D). To probe whether differential CD99 expression could still be of relevance in the animal model of MS, for example by influencing BBB transmigration, we performed an EAE by active immunization against MOG_35-55_ peptide with these animals for 30 days. Neither did we observe a difference in the clinical course, nor a change in body weight or the day of onset depending on the genotype (Fig. 4E). Furthermore, we analyzed immune cell infiltration in the CNS in the acute (day 15) and chronic phase (day 30) of EAE. Apart from macrophages, which were more abundant in the homozygous knockout mice compared to the WT littermates at day 15, none of the other immune cell subsets showed a difference (Figure S6D). However, we detected lower CD99 expression on peripheral T cells during EAE compared to healthy mice, while CNS-infiltrating T cells showed higher CD99 expression in acute EAE (day 15) (Figure S6E). To probe whether upregulation of other adhesion molecules could compensate for the loss of CD99 in BBB transmigration, we analyzed surface expression of CD99L2, VLA-4α, VLA-β and LFA-1α of αβ T cells isolated from the CNS, spleen and lymph nodes of WT, heterozygous or homozygous knockout mice (*n* = 6 per group), but did not detect any differences (Figure S6F). Taken together, these findings demonstrate that although CD99 surface expression levels are stepwise decreased in the three genotypes of our newly generated mouse line and thus might reflect the expression pattern in humans, we did not detect an impact on T cell function in vitro and EAE in vivo. Sex-specific surface expression pattern of CD99 and its functional consequences seem to be a species-specific trait in humans and can therefore not be analyzed further in the mouse model.

### CD99 is induced in primary human naïve T cells upon stimulation

**Fig. 5:**
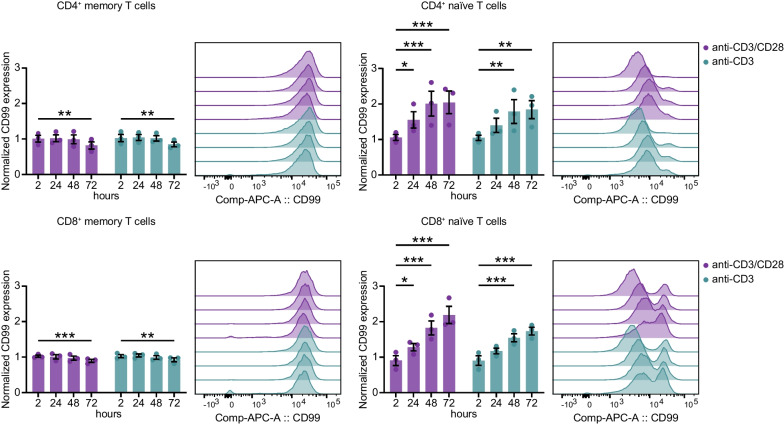
CD99 induction in human naïve T cells upon stimulation. Cryopreserved PBMCs from healthy controls (*n* = 3 females) were treated with anti-CD3 and anti-CD28 mAbs and cultured for 72 h. CD99 surface protein expression of memory and naïve CD4^+^ T cells (upper row) and CD8^+^ T cells (lower row) was analyzed by flow cytometry. Expression levels are normalized to the individual uncultured control. Data are shown as mean ± SEM. Statistics: two-way ANOVA with Dunnett post-hoc; *P < 0.05; **P < 0.01; ***P < 0.001

Having shown that the mouse is not a suitable model organism to study sex-specific CD99 function, we focused on human T cell function in in vitro assays. While some studies describe CD99 to be involved in T cell activation [35, 46] and other studies attribute an inhibitory role [34], the regulation of CD99 itself upon T cell stimulation has not been investigated. To further explore the role of CD99 in human T cell regulation, we first analyzed CD99 dynamics upon T cell stimulation. We detected an induction of CD99 in anti-CD3/anti-CD28 stimulated naïve CD4^+^ and CD8^+^ T cells in a time-dependent manner over 72 h. However, in memory CD4^+^ and CD8^+^ T cells, CD99 levels remained stable for 48 h and were even slightly decreased after 72 h (Fig. 5; Figure S7A), while upregulation of CD69 on naïve and memory T cells confirmed proper T cell activation of both subsets. We further used a blocking anti-CD99 antibody (mAb clone hec2) in these assays to probe whether this could have an influence on T cell activation. However, we did not observe altered CD69 expression levels in these conditions (Figure S7B), while it has previously been shown that T cell proliferation can be inhibited by CD99 blockade. Together, these results indicate a rapid induction of CD99 on the cell surface of naïve T cells after primary activation until eventually they become memory T cells with no further regulation of CD99 upon re-activation. This suggests CD99 levels to rather reflect a trait of memory T cells than a state.

### CD99 blockade inhibits cell proliferation in primary human T cell monocultures

**Fig. 6:**
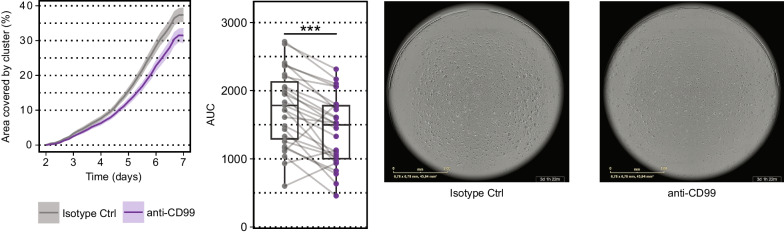
CD99 blockade inhibits proliferation in human T cells. Anti-CD3 and anti-CD28 stimulated cryopreserved T cells from 27 healthy controls (*n* = 12 females and *n* = 15 males) were treated with anti-CD99 mAb (clone HCD99) or the respective isotype control antibody and proliferation was tracked by cluster formation in the IncuCyte^®^ for 7 days. Data are shown as mean ± SEM. Statistics: Wilcoxon matched-pairs signed rank test; *P < 0.05; **P < 0.01; ***P < 0.001

It has previously been shown that anti-CD99 (mAb clone MT99/3) treatment inhibits T cell proliferation, but this inhibitory effect requires cell-to-cell contact between monocytes and lymphocytes, while soluble mediators produced by monocytes were insufficient to mediate hypo-responsiveness in T cells [45]. To probe whether this inhibition of proliferation by CD99 blockade can be mediated solely to CD99 signaling in T cells, we performed a proliferation assay with T cells in a monoculture. We stimulated freshly isolated T cells with anti-CD3/anti-CD28, added an anti-CD99 mAb (clone HCD99) and monitored proliferation by cluster formation over seven days. T cell clusters started to form at day two with a steady increase of their cluster size until eventually almost 40% of the analyzed area was covered by clusters at day seven. Notably, CD99 blockade led to an inhibition of T cell proliferation compared to isotype control-treated T cells (Fig. 6). Next, we asked whether expression differences in female and male T cells have a functional impact and analyzed the same samples by sex, but no significant difference in inhibition of proliferation by anti-CD99 treatment was observed (Figure S8). Together, these data show that specific blockade of CD99 in T cell monocultures is sufficient to inhibit cluster formation and proliferation, but that the effect of CD99 blockade is not different in a small sample size of women and men.

### Rescue of proliferation inhibition by CD99 reintroduction in T cells in vitro

**Fig. 7:**
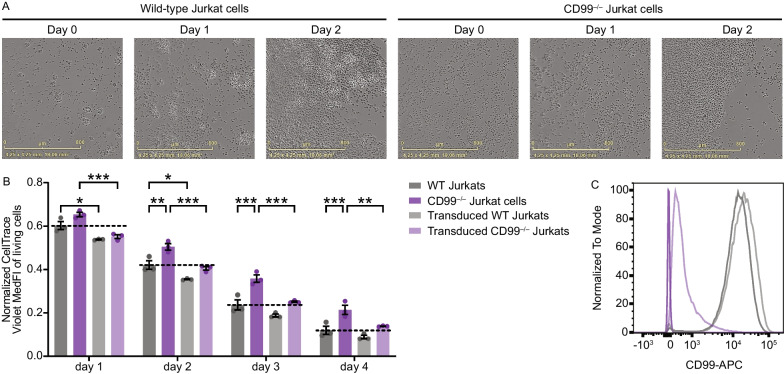
Rescue of proliferation inhibition by CD99 reintroduction in Jurkat T cells. **A** WT or *CD99*-deficient Jurkat T cells were tracked by cluster formation in the IncuCyte^®^ for 2 days. **B** Cell proliferation in WT Jurkat T cells, *CD99*-deficient Jurkat T cell clone and its rescued phenotype by lentiviral reintroduction of CD99 (*n* = 3 biological replicates with 3 technical replicates each). **C** CD99 surface expression of WT Jurkat T cells, *CD99*-deficient Jurkat T cells and their rescued phenotype as analyzed by flow cytometry. Data are shown as mean ± SEM. Statistics: **B** two-way ANOVA with Tukey post-hoc; *P < 0.05; **P < 0.01; ***P < 0.001

To mechanistically decipher our observation of specific T cell proliferation inhibition by CD99 blockade we next generated *CD99*-deficient Jurkat T cells. With this model we further aimed to probe the reported role of homophilic interaction in cell aggregation [26, 47]. Indeed, *CD99* deficiency in Jurkat T cells led to diminished cell aggregation and cluster formation compared to wildtype Jurkat T cells (Fig. 7A). To corroborate that reduced cluster formation has an impact on proliferation, we performed proliferation assays with fluorescently labelled dyes and detected a reduction of cell proliferation of *CD99*-deficient Jurkat T cells compared to wildtype Jurkat T cells in a time-dependent manner over four days (Fig. 7B). Moreover, this phenotype could be rescued by reintroduction of CD99 by lentiviral transduction with cell proliferation almost reaching the level of wildtype Jurkat T cells (Fig. 7B). As CD99 expression in transduced Jurkat T cells did not reach the same level as in wildtype cells (Fig. 7C), we hypothesized that there is a threshold of CD99 expression that is sufficient for the initiation of cell aggregation and thus proliferation, but not sufficient to maintain the same extent of proliferation as in wildtype cells over time. Notably, increasing CD99 surface expression levels in wildtype Jurkat T cells by lentiviral transduction even enhanced cell proliferation in those cells compared to unmodified wildtype Jurkat T cells (Fig. 7B,C). Together, these findings demonstrate a crucial role of homotypic CD99 interaction in cell aggregation and proliferation.

## Discussion

Sex is a critical biological variable in development and pathogenesis of autoimmune diseases including MS [1–4]. Here, we show that transcriptional regulation by both sex chromosomes, but not hormonal regulation, leads to sex-specific CD99 surface expression on immune cells, which is partially lost in the CSF of MS patients. However, these sex differences are human-specific as we do not see sexually dimorphic regulation in mice. Considering CD99 impact on T cell regulation, our findings pronounce the necessity of including sex as an important factor on phenotypical outcome in immune responses in women and men.

We found that *CD99* mRNA expression in the spleen and CD99 protein levels on the surface of T cells, pDCs and NK cells were higher in men. This sexual dimorphism is most likely explained by full CD99 expression from the pseudoautosomal region of both, the X and Y chromosome in men, while in women CD99 expression from one X chromosome is reduced due to XCI. Although *CD99* can escape XCI [30], it appears that transcription from the inactivated X chromosome cannot compensate for the Y-encoded expression. A possible reason why we do not see a sexual dimorphism of CD99 expression in other subsets like cDCs, B cells and monocytes could be cell-specific regulation of XCI, as has recently been shown for B cell-specific XIST complexes driving cell-type-specific diversification [48]. In general, XCI escapees can be classified as ‘constitutive’ or ‘facultative’ [49]. Constitutive escapees are expressed in most cell types, whereas facultative escapees appear to be silenced and then reactivated and can be lineage specific and differ between individuals, developmental stages or ages.

Supporting the hypothesis of incomplete XCI as driver of the sexual dimorphism in CD99 expression, we could not find evidence for hormonal regulation. The classical perspective of androgen action on gene regulation is that testosterone is intracellularly converted into its more potent bioactive form DHT, which binds to the androgen receptor (AR) with high affinity [50]. This results in removal of the heat-shock protein and binding to importin-α to translocate into the nucleus, where receptor dimers bind to androgen response elements (AREs) in promoter regions of target genes to enhance or repress transcription. Analysis of AREs in *CD99* promoter regions need further investigation. However, our in vitro data proposes no interaction between androgen signaling and CD99 levels. This is corroborated by our ex vivo human data, although the sample size of trans men individuals was low. These findings suggest that incomplete XCI accounts for sex-specific expression patterns of CD99 and that Y-encoded transcription is insufficiently compensated by transcription from the inactivated X chromosome.

Studying sex differences in humans is challenging. To investigate functional consequences in more detail, we aimed to transfer our findings into the mouse model. Though, CD99 surface expression did not show sex-specific differences in T cells; these findings could at least be partially attributed to the unique genetical features of *CD99*. In humans, together with *Xga* and CD99 antigen-like 2 (*CD99L2*), which are also located in the pseudoautosomal region, CD99 composes a family of proteins, for which no homology to any other known family is reported [51]. Additionally, CD99 homologs were only found in primates and the mouse ortholog (D4) shows only 46% protein homology with human CD99 and is located on chromosome 4 [52]. It is thought that the *CD99* orthologous genes of carnivores and artiodactyls should be located in the pseudoautosomal region and that human and mouse *CD99* developed differently with the translocation of mouse *Cd99* to an autosome after the divergence of rodents and other non-primate eutherians [52]. With mouse *Cd99* located on an autosome, the absence of sexual dimorphism of CD99 expression in mice is plausible and further corroborates differential expression from the activated or inactivated X chromosome and/or the Y chromosome as driver of the sexual dimorphism in CD99 expression in humans instead of hormonal regulation.

To circumvent the absence of sexual dimorphism in mice, but still use a mouse model for mechanistic analyses of consequences of differential CD99 expression, we newly generated *Cd99*-deficient mice and used heterozygous littermates as female mimics. However, we did not observe any differences in T cell proliferation between WT, homozygous or heterozygous knockout mice, which suggests that CD99 is not involved in mouse T cell activation or that in the knockout condition compensatory mechanisms are able to fully restore T cell function. Additionally, although anti-CD99 treatment was shown to ameliorate the disease course in EAE [28] and CD99 was higher expressed in CNS-infiltrating T cells, we did not observe any impact of genetic ablation of CD99 on clinical symptoms or body weight change in EAE. This discrepancy might be partially explained by compensatory mechanisms. However, we detected no differences in the expression of other adhesion molecules, such as LFA-1, VLA-4 or CD99L2, another CD99 paralog in mice, that could compensate for the loss of CD99. Although *Cd99l2* shares 45% homology with the human *CD99* gene and 81% homology with human *CD99L2* gene, there is only limited overlap in function [51]. Nevertheless, investigating CD99L2 and its potential compensatory role in *Cd99*-deficient mice might be worthwhile as an interaction between these two molecules has been shown [53]. Moreover, anti-CD99L2 antibodies blocked neutrophil recruitment into the inflamed peritoneum and cremaster [54–56] and genetic inactivation of the *Cd99l2* gene revealed its importance for the entry of lymphocytes and neutrophils into inflamed tissues [57]. Of note, it has been shown that conditional Tie-2-Cre driven gene inactivation of *Cd99l2* inhibits leukocyte entry into the CNS during MOG_35-55_-induced EAE and alleviates severity of the disease [58].

Similar to human healthy individuals, we observed increased CD99 expression in male MS patients. Because of its association with T cell activation and transmigration, we hypothesized increased CD99 expression in MS patients, which could be a driver of an overall higher inflammatory state, but we did not find any significant differences between MS patients and healthy individuals in the peripheral blood. However, in CSF samples we detected increased CD99 levels on T cells compared to peripheral blood in every individual regardless of disease state. This is in accordance with a recent single-cell analysis of blood and CSF leukocytes that revealed CD99 to be more abundant on CD4^+^ and CD8^+^ T cells in the CSF [59]. Of note, in our cohorts male MS patients showed lower CD99 levels on CD4^+^ T and CD8^+^ T cells in comparison to male patients with non-neuroinflammatory diseases. This could be explained by a suppression or lack of induction of CD99 through inflammatory processes in the CNS to prevent hyper-inflammation. However, we could not detect regulation of CD99 expression in response to TCR activation in memory cells in vitro. Alternatively, CD99 decrease in the CSF of MS patients could also be due to CD99 downregulation after recognition of its ligand resulting in a residency of activated T cells at the site of inflammation. In the context of altered CD99 expression on T cells in the CSF, differential expression of CD99 ligands could be an additional factor contributing to functional differences between females and males as it influences the homeostatic equilibrium. Besides its homophilic interaction [26], the existence of other CD99 ligands has been proposed [45, 46, 60]; such as the GDF6 prodomain, which maintains Ewing sarcoma growth [61] or the paired immunoglobulin-like receptors (PILRs), capable of binding mouse CD99 [62, 63]. However, there is currently not much known about their sex-specific differential expression in males and females as well as their expression in different organs and cell types. Another explanation for reduced CD99 expression in the CSF in MS could be that during CNS inflammation, CD4^+^ T cells with lower CD99 expression are able to transmigrate from the periphery to the CNS compartment leading to an overall lower CD99 expression in the CNS due to a leakier BBB. CD99 involvement in T cell transmigration has been extensively studied [54, 64–69], but none of these studies took sex-specific differences into account. We focused on effects of CD99 blockade and genetic ablation on T cell activation and in turn proliferation, but studying the role of CD99 in transmigration in a sex-specific manner would shed light on further consequences of the sexual dimorphism in CD99 expression.

We found that CD99 expression is induced in naïve T cells upon TCR activation, while memory T cells did not regulate CD99 expression in response to activation. Induction of CD99 upon T cell activation stresses its importance in T cell regulation and indicates that CD99 acts as a co-stimulatory factor in T cell activation [35, 64]. Engagement of CD99 and suboptimal anti-CD3-induced T cell activation was comparable to that obtained with anti-CD3/anti-CD28 [35] and enhanced the expression of several T cell activation markers and cytokines on anti-CD3-stimulated T cells [46, 70]. Additionally, we observed inhibition of homotypic T cell clustering by anti-CD99 mAb, which indicates a crucial role of CD99 for T cell clustering as a prerequisite for T cell activation. CD99 might be essential in maintaining T cell clusters via its homophilic interaction as it has been described for LFA-1/ICAM-1 [71]. Along that line, CD99 has been reported to form complexes with MHC-I, MHC-II and tetraspanin CD81 and is associated to the formation of the immunologic synapse [34, 72] as well as translocation of TCR complexes to the lipid raft [35]. Our data showing inhibition of T cell proliferation in primary human T cells by anti-CD99 treatment in vitro complements previous findings. While agonistic mAb treatment (clone 3B2/TA8) has been shown to induce proliferation in resting peripheral blood T cells [36], other studies found an inhibition of T cell proliferation with anti-CD99 mAb (clone MT99/3) in PBMCs [34] or in a co-culture of T cells and monocytes [45]. Additionally, by CD99 reintroduction and subsequent rescue, we could attribute these findings to CD99 properties. Considering all these findings, one can speculate that a lower expression of CD99 in women is a counterregulatory mechanism to compensate for an overall lower activation threshold of female T cell activation [1].

## Perspectives and significance

Taken together we were able to show that CD99 is regulated in a sex- and species-specific manner and functionally contributes to T cell costimulation in humans stressing the relevance for stratification of immunological data by sex. Differential regulation of CD99 on CSF T cells in female and male MS patients might hint to a functional sex-specific involvement of CD99 in MS pathogenesis, which warrants further investigation.

### Supplementary Information

Additional file1 (PDF 6235 KB)

Additional file2 (XLSX 4738 KB)

Additional file3 (XLSX 50 KB)

Additional file4 (XLSX 4770 KB)

Additional file5 (XLSX 4674 KB)

## Data Availability

All data generated or analyzed during this study are included in this published article and are available from the corresponding author upon request.
