# Supplementary material for: Sex- and species-specific contribution of CD99 to T cell costimulation during multiple sclerosis
Source: Biol Sex Differ. 2024 May 15;15:41. doi: 10.1186/s13293-024-00618-y (PMC11097467; doi:10.1186/s13293-024-00618-y)
Supplement: Supplementary file 1 — Additional file1 (PDF 6235 KB) [file 13293_2024_618_MOESM1_ESM.pdf]

SUPPLEMENTARY INFORMATION

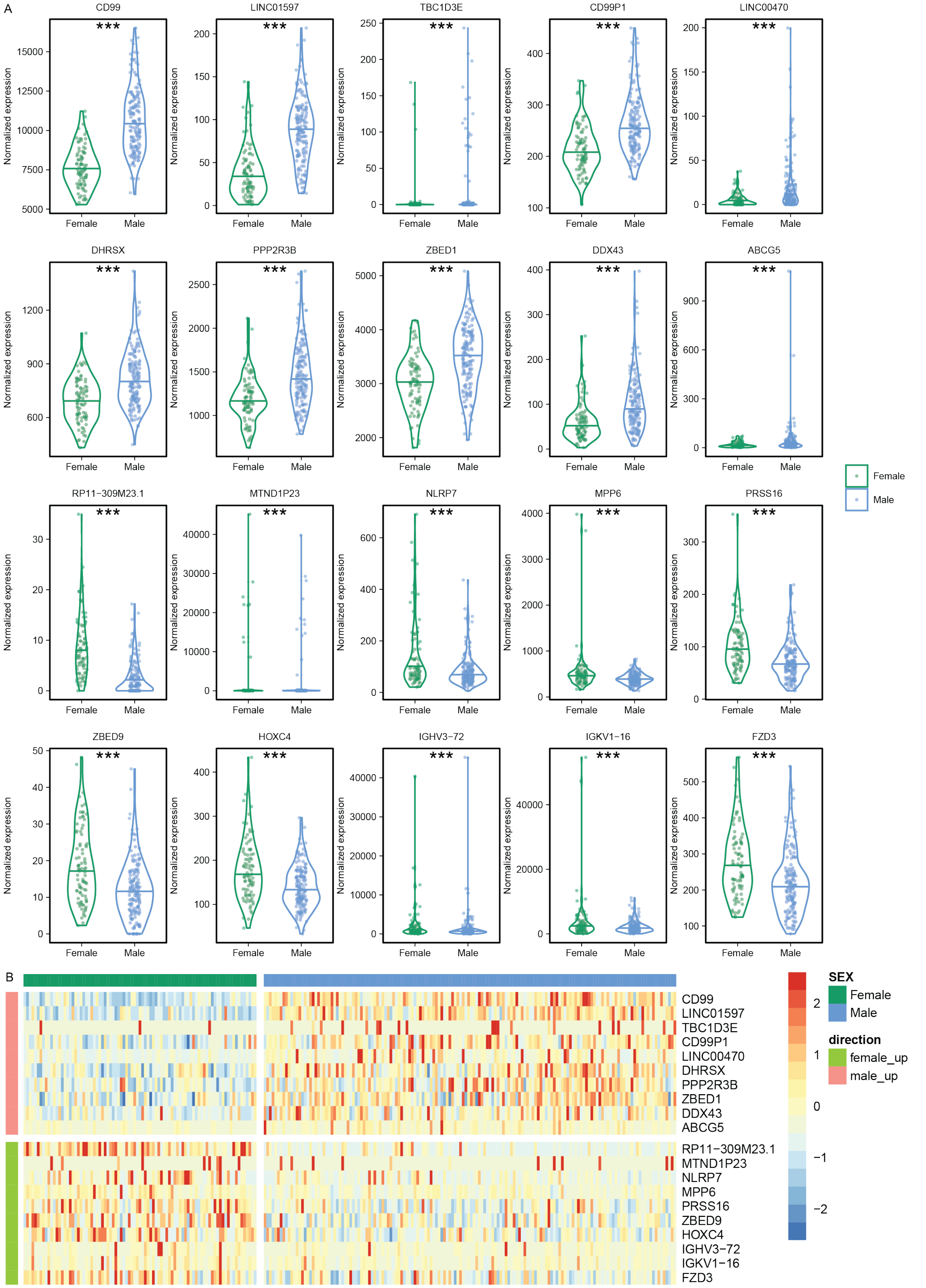

Figure S1. Differentially expressed genes in males and females. (**A**) mRNA expression levels of top 20 sex-specific genes in spleen samples (n = 87 women and 154 men) from the Genotype-Tissue Expression (GTEx) dataset. (**B**) Individual gene expression levels from the same dataset as shown in a heatmap. Data are shown as violin plots including median. Statistics: DESeq2 false discovery rate-adjusted P value; *P < 0.05; **P < 0.01; ***P < 0.001.

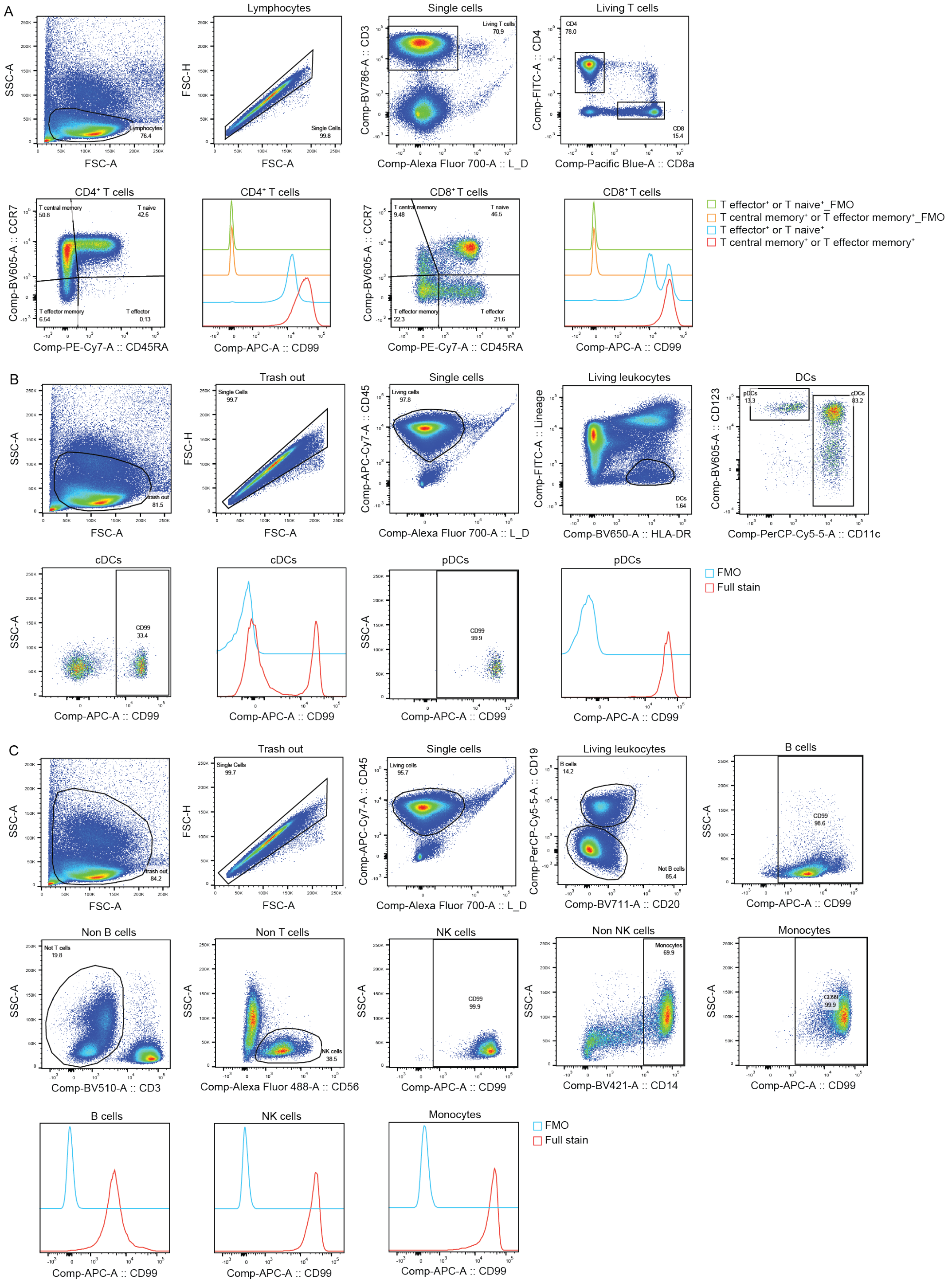

Figure S2. Identification of immune cell subsets in PBMC cohort. (**A-C**) Representative gating strategy for identification of (**A**) T cells, (**B**) dendritic cells as well as (**C**) B cells, NK cells and monocytes.

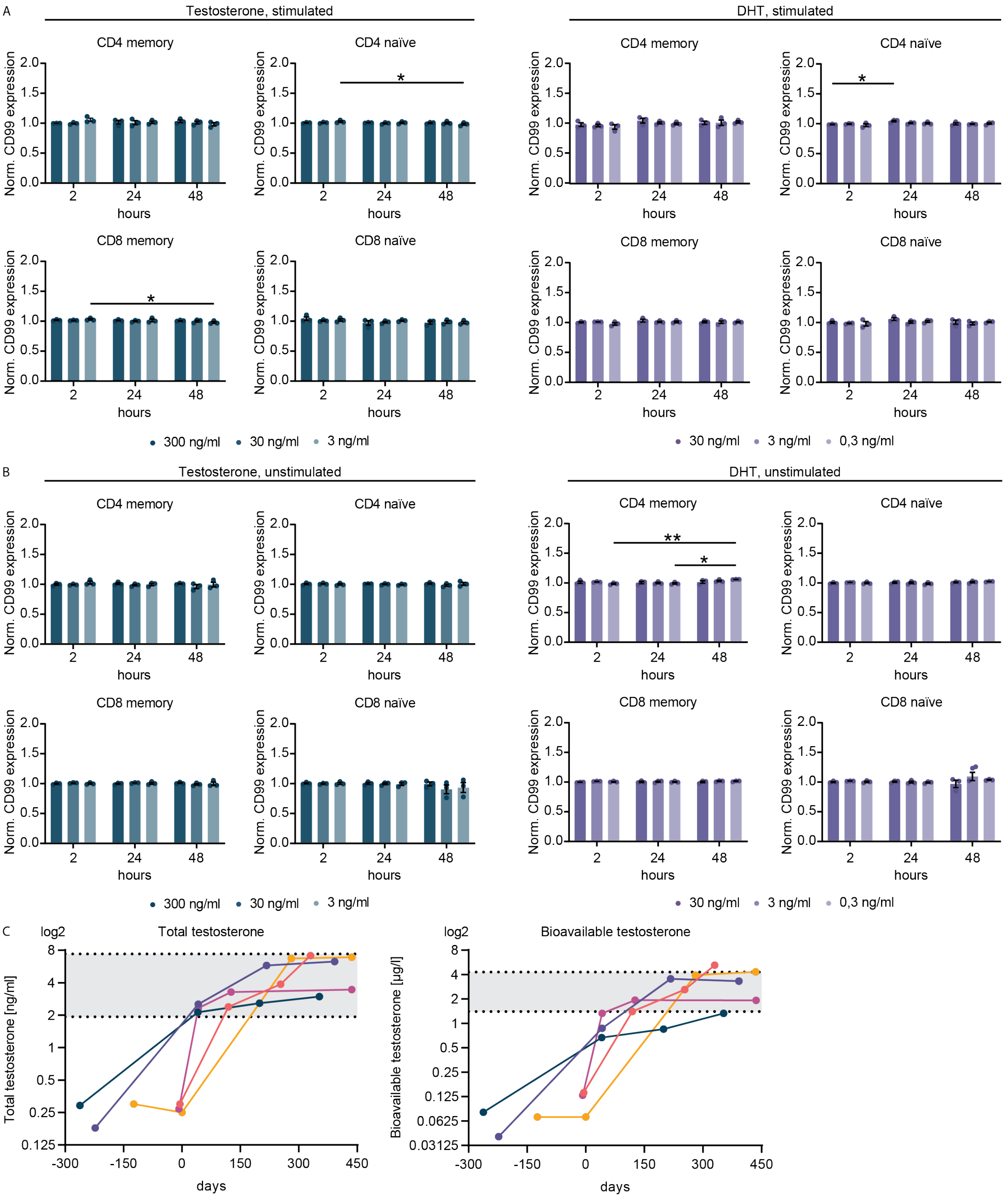

Figure S3. Testosterone impact on CD99 expression of human T cells. (**A**) CD99 surface expression on T cells of anti-CD3 and anti-CD28 stimulated cryopreserved PBMCs (*n* = 3 males) treated with different concentrations of testosterone or dihydrotestosterone. Expression values are normalized to the individual vehicle control for every time point. (**B**) CD99 surface expression on T cells of unstimulated cryopreserved PBMCs (*n* = 3 males) treated with different concentrations of testosterone or dihydrotestosterone. Expression values are normalized to the individual vehicle control for every time point. (**C**) Total testosterone and bioavailable testosterone serum levels of trans men (*n* = 5). The male serum reference range is indicated by the grey shade (total testosterone: 1.93-7.4 ng/mL, bioavailable testosterone: 1.4-4.3 µg/l). Each color represents the same individual donor. Data are shown as mean ± SEM. Statistics: (A, B) two-way ANOVA with Tukey post-hoc; **P* < 0.05; ***P* < 0.01; ****P* < 0.001.

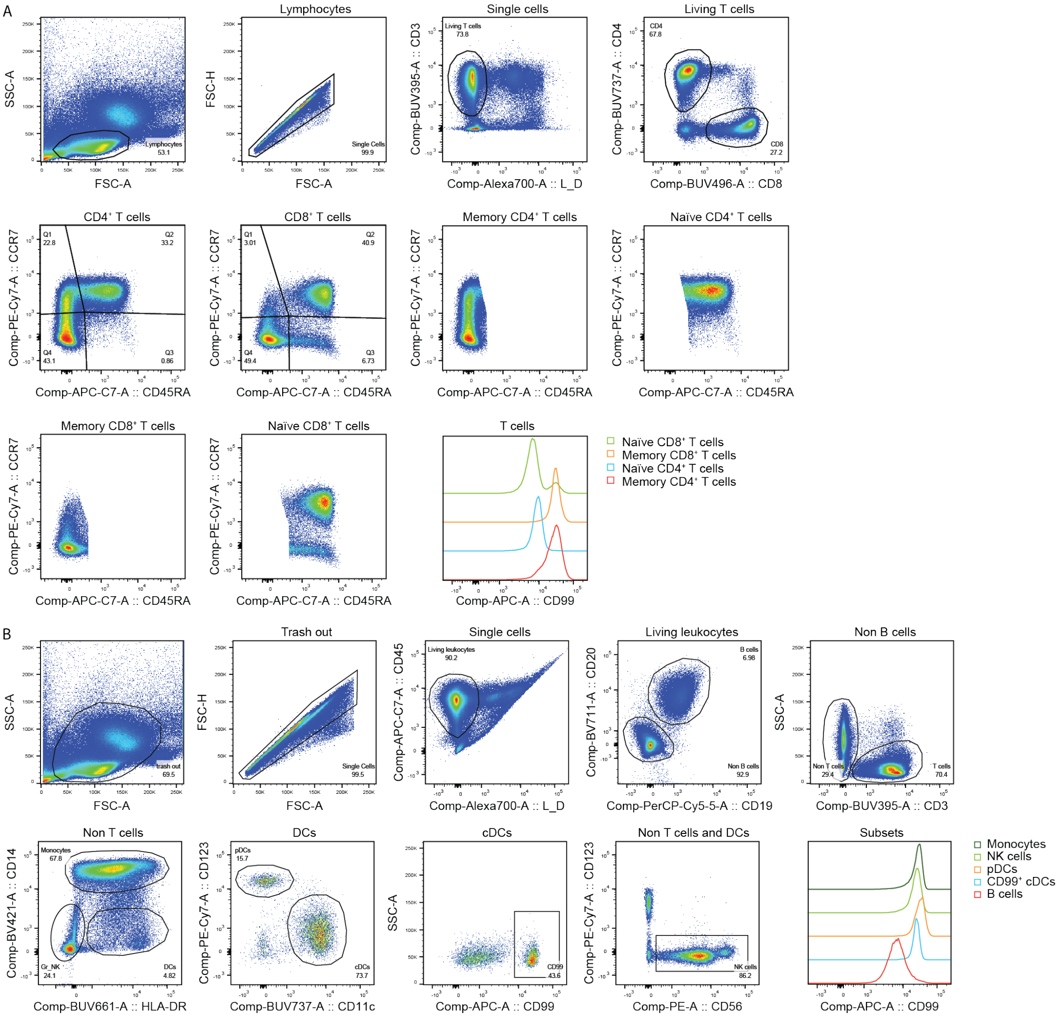

Figure S4. Identification of immune cell subsets in trans men cohort. (**A-C**) Representative gating strategy for identification of (**A**) T cells, (**B**) dendritic cells, B cells, NK cells and monocytes.

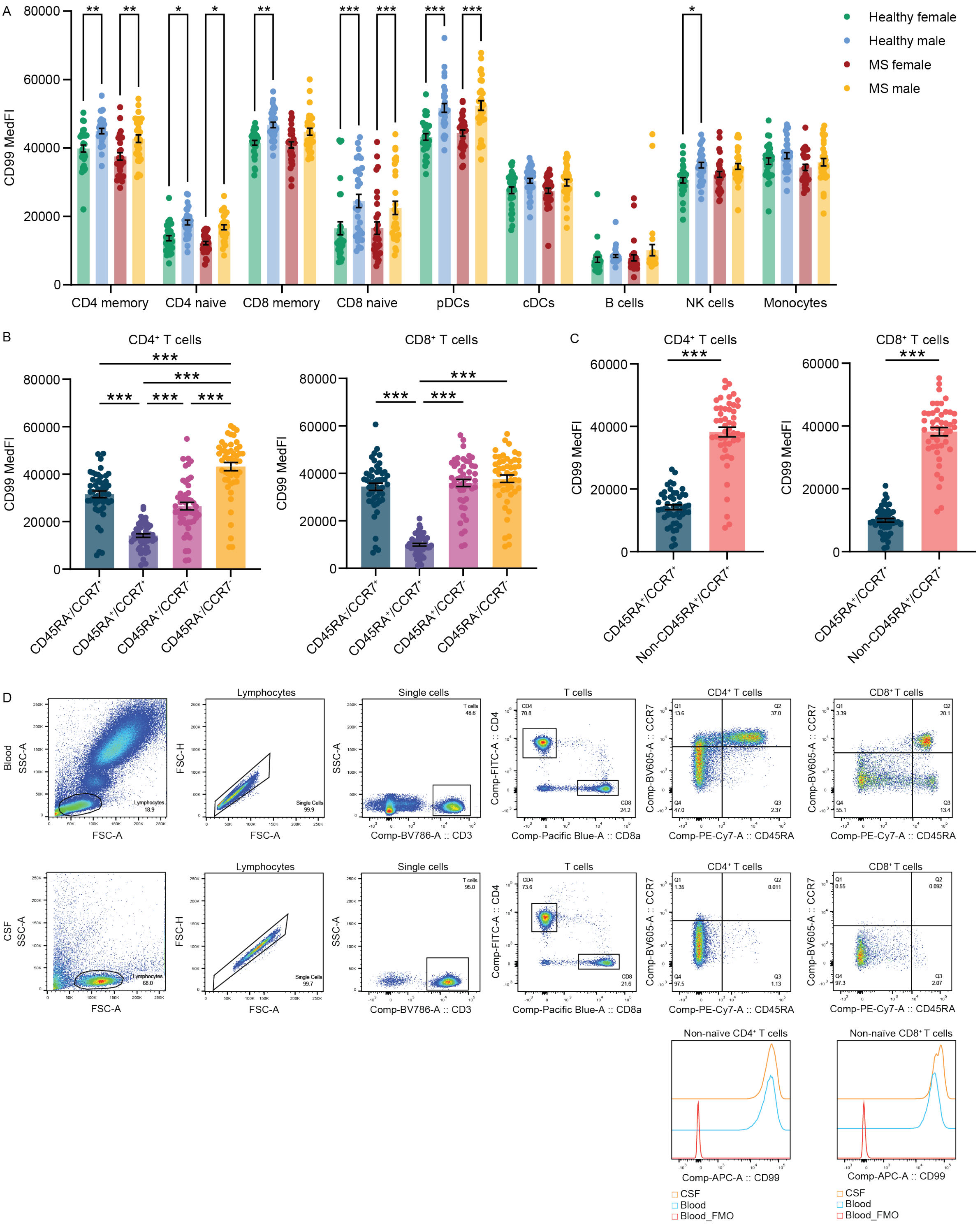

Figure S5. CD99 expression in immune cells from healthy individuals and MS patients. (**A**) CD99 surface protein expression on subsets of cryoconserved peripheral blood mononuclear cells (PBMCs) from male (n = 30) and female (n = 30) healthy individuals and MS patients as analyzed by flow cytometry. (**B**) CD99 expression levels on freshly isolated $CD4^+/CD8^+$ central memory T cells (Q1: $CD45RA^-CCR7^+$), naïve T cells (Q2: $CD45RA^+CCR7^+$), effector T cells (Q3: $CD45RA^+CCR7^-$) and effector memory T cells (Q4: $CD45RA^-CCR7^-$) in a representative individual as analyzed by flow cytometry. (**C**) Comparison of CD99 expression levels of the same individual on freshly isolated naïve T cells (Q2: $CD45RA^+CCR7^+$) and "non-naïve" T cells (Q1: $CD45RA^-CCR7^+$, Q3: $CD45RA^+CCR7^-$ and Q4: $CD45RA^-CCR7^-$ combined). (**D**) Representative gating strategy for the characterization of T cell subsets in freshly isolated blood or CSF samples. Data are shown as mean ± SEM. Statistics: (A) two-way ANOVA with Tukey post-hoc; (B) Friedman test with Dunn post-hoc; (C) Wilcoxon matched-pairs signed rank test; *P < 0.05; **P < 0.01; ***P < 0.001.

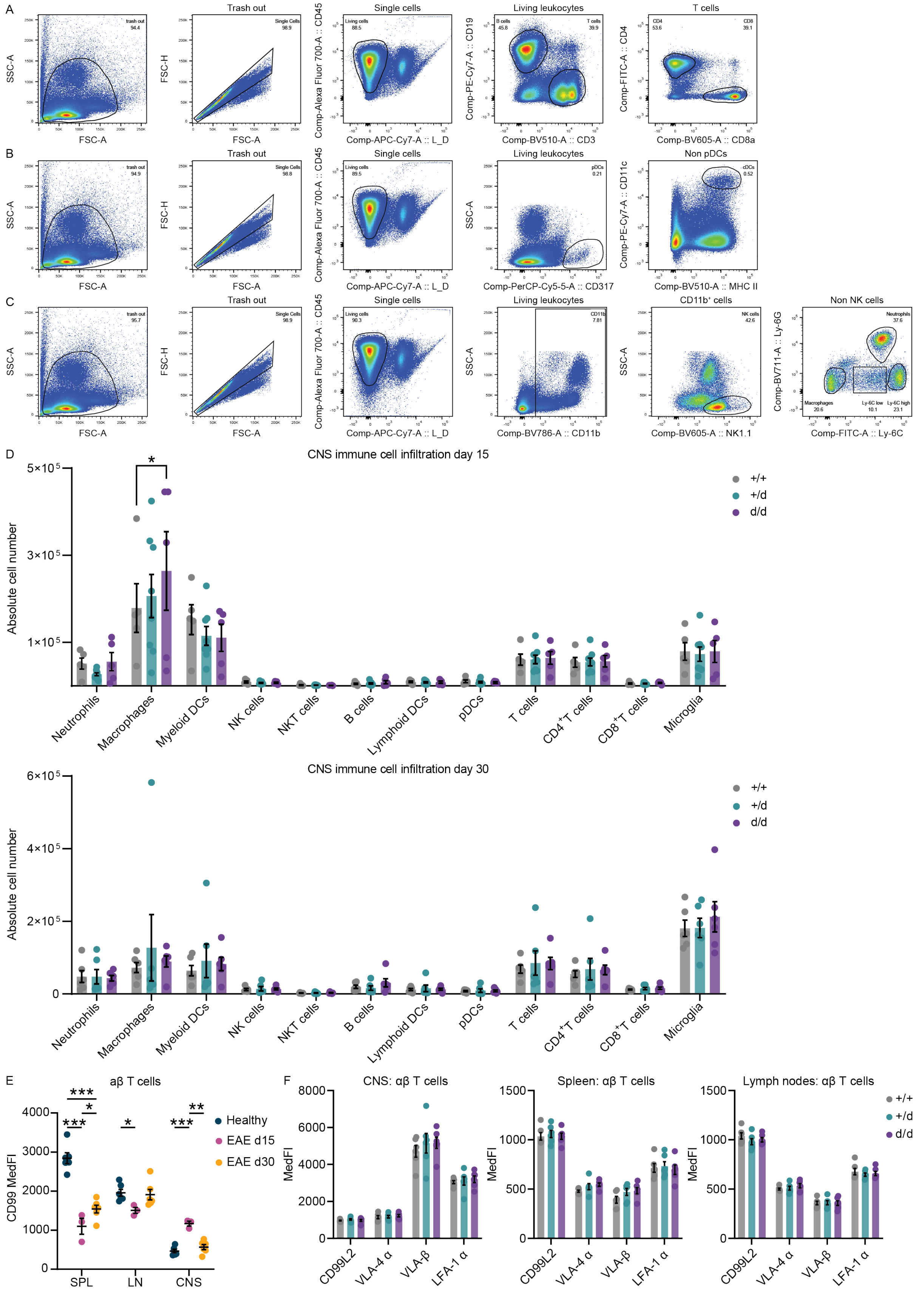

Figure S6. Immune cell phenotyping of C57B6/J WT and *Cd99*-deficient mice. (**A-C**) Representative gating strategy for identification of (**A**) T cells, (**B**) dendritic cells and (**C**) innate immune cells. (**D**) Immune cell infiltration during EAE at acute phase (upper panel, day 15; *n* = 5 male d/d, *n* = 2 female +/d, *n* = 6 male +/d, *n* = 5 male +/+) and chronic phase (lower panel, day 30; *n* = 3 females and 3 males per group). (**E**) CD99 surface protein expression of T cells in the spleen (SPL), lymph nodes (LN) and CNS of WT mice (*n* = 6 healthy +/+, *n* = 3 EAE day 15 +/+, *n* = 6 EAE day 30 +/+) as analyzed by flow cytometry. (**F**) Surface expression of CD99L2, VLA-4α, VLA-β and LFA-

1α of $\alpha\beta$ T cells isolated from the CNS, spleen and lymph nodes of WT, heterozygous or homozygous knockout mice (*n* = 4 female d/d, *n* = 2 male d/d, *n* = 3 female +/d, *n* = 3 male +/d, *n* = 3 female +/+, *n* = 3 male +/+) as analyzed by flow cytometry. Data are shown as mean ± SEM. Statistics: (D-F) two-way ANOVA with Tukey post-hoc; $^{*}P < 0.05$; $^{**}P < 0.01$; $^{***}P < 0.001$.

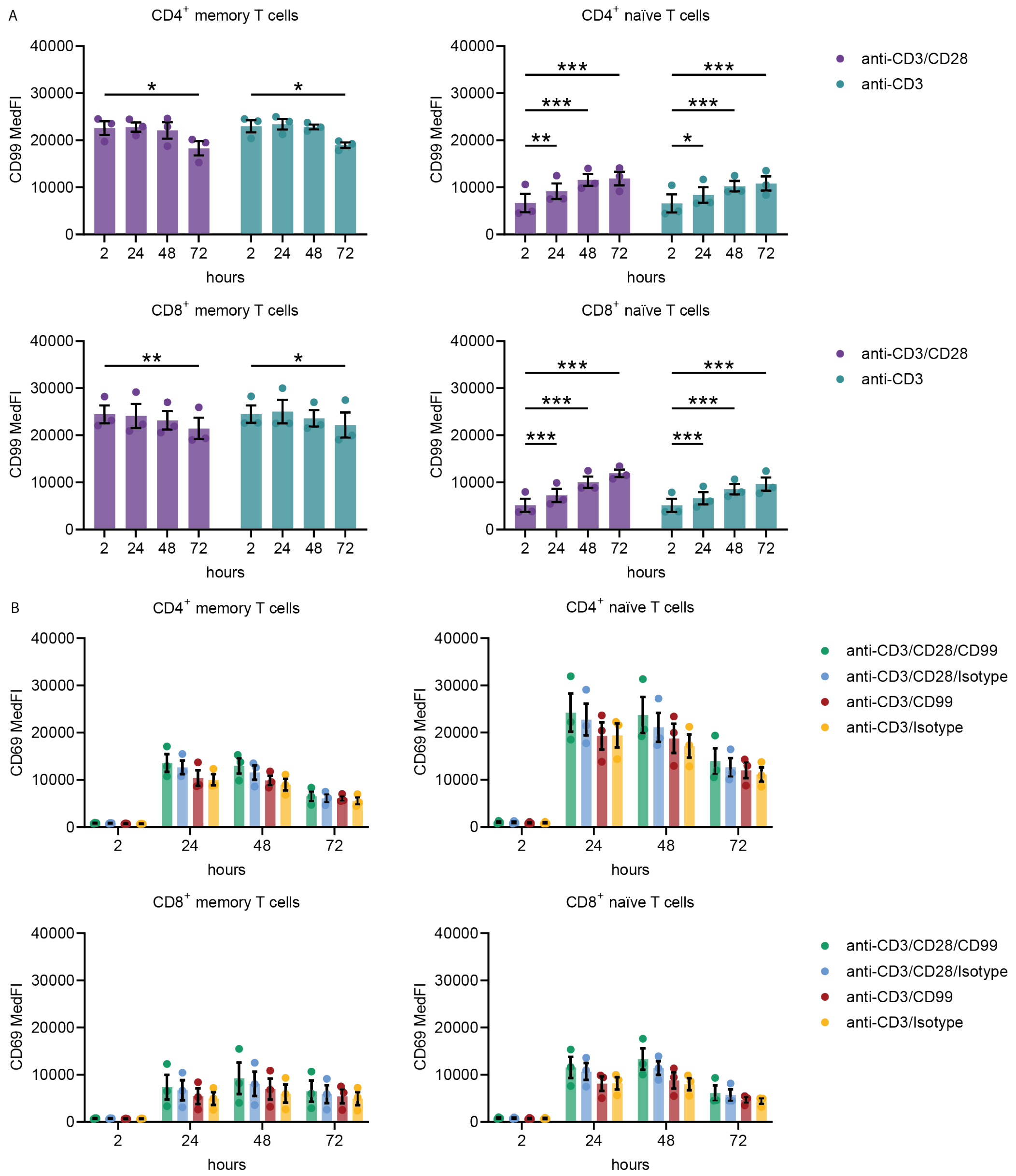

Figure S7. CD99 dynamics in human T cells upon stimulation. Cryopreserved PBMCs from healthy controls (*n* = 3 females) were treated with anti-CD3 and anti-CD28 mAbs and cultured for 72 hours. (**A**) Raw CD99 expression values of memory and naïve T cells was analyzed by flow cytometry. (**B**) Raw CD69 expression values of memory and naïve T cells as a marker for T cell activation analyzed by flow cytometry. Data are shown as mean ± SEM. Statistics: (A) two-way ANOVA with Dunnett post-hoc; (B) two-way ANOVA with Tukey post-hoc; **P* < 0.05; ***P* < 0.01; ****P* < 0.001.

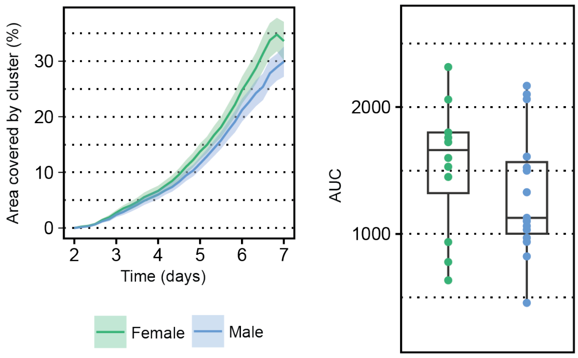

Figure S8. Sex-specific analysis of anti-CD99 treatment on cell proliferation in human T cells. Anti-CD3 and anti-CD28 stimulated cryopreserved T cells from 27 healthy controls (*n* = 12 women and *n* = 15 men) were treated with anti-CD99 mAb (clone HCD99) or the respective isotype control antibody and proliferation was tracked by cluster formation in the IncuCyte® for 7 days. Data are shown as mean ± SEM. Statistics: Wilcoxon matched-pairs signed rank test; *$P < 0.05$; **$P < 0.01$; ***$P < 0.001$.

Table S1. Characteristics of MS patients and healthy individuals included in CD99 surface expression analysis of PBMCs.

	n	**% female**	**Age (years)**	**Disease duration (years)**	**Median EDSS[1] ± IQR**
RRMS[2]	60	50	33.3 ± 9.8	5.1 ± 5.7	1.5 ± 2.0
HI[3]	60	50	33.0 ± 9.7	NA[4]	NA

[1]Expanded disability status scale; [2]Relapsing remitting MS; [3]Healthy individuals; [4]Not applicable. Data are presented as mean ± SD unless otherwise indicated.

Table S2. Characteristics of MS patients and non-neuroinflammatory disease patients included in CD99 surface expression analysis of blood and CSF samples.

	n	**% female**	**Age (years)**	**Disease duration (years)**	**Median EDSS[1] ± IQR**
RRMS[2]	33	73%	33.8 ± 9.8	1.6 ± 2.4	1.5 ± 1.0
NND[3]	16	69%	41.3 ± 13.2	NA[4]	NA

[1]Expanded disability status scale; [2]Relapsing remitting MS; [3]Non-neuroinflammatory disease patients; [4]Not applicable. Data are presented as mean ± SD unless otherwise indicated.

Table S3. Characteristics of healthy individuals included in CD99 surface expression analysis of PBMCs in trans men cohort.

n	Mean age of treatment start (years)	Median injection date TP1[1] (months)	Median injection date TP2 (months)	Treatment
**5**	23.3 ± 2.9	6.7 ± 0.4	11.9 ± 0.7	1000 mg testosterone undecanoate i.m.

[1]Time point. Data are presented as means (range; SD) unless otherwise indicated.

Table S4. Antibodies used for flow cytometry.

Antigen	Clone	Supplier
**PBMC cohort**		
CD4	RPA-T4	BioLegend
CD161	HP-3G10	BioLegend
CD45RA	HI 100	BioLegend
CD8a	HIT8a	BioLegend
CD197 (CCR7)	G043H7	BioLegend
CD3	OKT3	BioLegend
Anti-Human Lineage (CD3,CD14,CD19,CD20,CD56)	UCHT1, HCD14, HIB19, 2H7, HCD56	BioLegend
CD11c	3.9	BioLegend
CD16	3G8	BioLegend
CD304	12C2	BioLegend
CD123	6H6	BioLegend
HLA-DR	G46-6	BD Bioscience
CD45	HI30	BioLegend
CD56	B159	BD Bioscience
CD19	SJ25C1	BioLegend
CD14	HCD14	BioLegend
CD20	2H7	BD Bioscience
CD99	HCD99	BioLegend
**CSF cohort**		
CD4	RPA-T4	BioLegend
CD161	HP-3G10	BioLegend
CD45RA	HI 100	BioLegend
CD8a	HIT8a	BioLegend
CD197 (CCR7)	G043H7	BioLegend
CD3	OKT3	BioLegend
CD99	HCD99	BioLegend
**CD99 dynamics**		
CD3	SK7	BD Bioscience
CD8	RPA-T8	BD Bioscience
CD4	SK3	BD Bioscience
CD69	FN50	BioLegend
CD99	3B2/TA8	BioLegend
CD45RA	HI100	BioLegend
***in vitro* testosterone**		
CD3	SK7	BD Bioscience
CD8	RPA-T8	BD Bioscience
CD4	SK3	BD Bioscience

CD69	FN50	BioLegend
CD99	3B2/TA8	BioLegend
CD45RA	HI100	BioLegend
**Trans men cohort**		
CD3	SK7	BD Bioscience
CD8	RPA-T8	BD Bioscience
CD4	SK3	BD Bioscience
CD161	DX12	BD Bioscience
MR1 tetramer		NIH
CCR7	G043H7	BioLegend
CD99	3B2/TA8	BioLegend
CD45RA	HI100	BioLegend
CD16	3G8	BD Bioscience
HLA-DR	G46-6	BD Bioscience
CD11c	B-ly6	BD Bioscience
CD14	HCD14	BioLegend
CD20	2H7	BioLegend
CD19	SJ25C1	BioLegend
CD56	HCD56	BioLegend
CD123	6H6	BioLegend
CD45	HI30	BioLegend
**CD99 expression in C57BL6/J**		
CD11b	M1/70	BD Bioscience
TCRb	H57-597	BD Bioscience
MHC II	M5/114.15.2	BioLegend
CD19	6D5	BioLegend
CD4	GK1.5	BioLegend
CD8a	53-6.7	BioLegend
CD45	30-F11	BioLegend
NK1.1	PK136	BioLegend
CD11c	N418	BioLegend
CD317	927	BioLegend
Ly-6G	1A8	BioLegend
CD99	polyclonal	R&D
***CD99*-deficient mouse validation**		
CD11b	M1/70	BD Bioscience
B220	RA3-6B2	BD Bioscience
TCRb	H57-597	BD Bioscience
TCRgd	GL3	BD Bioscience
CD4	GK1.5	BioLegend

CD8	53-6.7	BioLegend
CD44	IM7	BioLegend
CD45	30-F11	BioLegend
CD99	polyclonal	R&D
**Mouse CFSE proliferation**		
CD4	GK1.5	BD Bioscience
TCRb	H57-597	BD Bioscience
CD8a	53-6.7	BioLegend
CD44	IM7	BioLegend
**EAE infiltration day 30**		
CD11b	M1/70	BD Bioscience
CD19	1D3	BD Bioscience
TCRb	H57-597	BD Bioscience
MHC II	M5/114.15.2	BD Bioscience
F4/80	BM8	BioLegend
NK1.1	PK136	BD Bioscience
CD8a	53-6.7	BioLegend
CD45	30-F11	BioLegend
Ly6G	1A8	BioLegend
CD11c	N418	BioLegend
CD317	927	BioLegend
CD99	polyclonal	R&D
**EAE infiltration day 15**		
CD11b	M1/70	BD Bioscience
CD19	1D3	BD Bioscience
TCRb	H57-597	BD Bioscience
MHC II	M5/114.15.2	BD Bioscience
F4/80	BM8	BioLegend
NK1.1	PK136	BD Bioscience
CD8a	53-6.7	BioLegend
CD45	30-F11	BioLegend
Ly6G	1A8	BioLegend
CD11c	N418	BioLegend
CD317	927	BioLegend
TCRgd	GL3	BD Bioscience
PD-1/CD279	29F.1A12	BioLegend
CD103	2E7	BioLegend
CD44	IM7	BioLegend
CD69	H1.2F3	BioLegend
CD4	GK1.5	BioLegend

CD99	polyclonal	R&D systems
**Cd99-deficient mice integrins**		
CD11b	M1/70	BD Bioscience
CD19	1D3	BD Bioscience
TCRb	H57-597	BD Bioscience
CD99l2	polyclonal	R&D systems
CD8a	53-6.7	BioLegend
CD11a	M17/4	BioLegend
CD44	IM7	BioLegend
CD99	polyclonal	R&D systems
CD29	HMb1-1	BioLegend
CD49d	R1-2	BioLegend
CD45	30-F11	BioLegend

Table S5. Primers, oligonucleotides and respective restriction sites used for the generation of CD99 vectors.

Abbreviation	Name	Full sequence
Primer_f_1	for_hsCD99_BamHI	TAG GGATCC GAT GGT GGT TTC GAT TTA TCC GAT G
Primer_r_1	rev_hsCD99_XbaI	TAG TCTAGA TTT CTC TAA AAG AGT ACG CTG AAC AGC
Oligo_f_1	LCv2-hsCD99-sgRNA01_FWD	CACCGCGGCGACCAGAACACCCAGC
Oligo_r_1	LCv2-hsCD99-sgRNA01_REV	aaacGCTGGGTGTTCTGGTCGCCGC
Oligo_f_2	LCv2-hsCD99-sgRNA02_FWD	CACCGATCCCCAAGAAACCCAGTGC
Oligo_r_2	LCv2-hsCD99-sgRNA02_REV	aaacGCACTGGGTTTCTTGGGGATC
Oligo_f_3	LCv2-hsCD99-sgRNA03_FWD	CACCGAAAGTCATCCCCTAAAAGAG
Oligo_r_3	LCv2-hsCD99-sgRNA03_REV	aaacCTCTTTTAGGGGATGACTTTC
Oligo_f_4	LCv2-hsCD99-sgRNA04_FWD	CACCGCACCTGAAACGCCATCCGCA
Oligo_r_4	LCv2-hsCD99-sgRNA04_REV	aaacTGCGGATGGCGTTTCAGGTGC
